# Links between Vaccination Fear-, Anxiety-, Alexithymia-, and Type D Personality-Related Vaccination Decisions: A Network Analysis in a Multicultural Sample

**DOI:** 10.3390/bs14090761

**Published:** 2024-08-29

**Authors:** Olga Malas, Nada Mallah Boustani, Mirko Duradoni, Dayo Omotoso, Asiye Şengül Avşar, Anastasiia Shyroka, Giulia Colombini, Maria Dolores Tolsá

**Affiliations:** 1 Department of Psychology, Sociology and Social Work, University of Lleida, Avinguda de l’Estudi General, 4, 25001 Lleida, Spain; 2 Faculty of Business and Management, Saint Joseph University, Mar Mikhael, Beirut 1104 2020, Lebanon; 3 Department of Education, Languages, Interculture, Literatures and Psychology, University of Florence, Via di San Salvi, 12, Building 26, 50135 Florence, Italy; 4 Department of Human Anatomy, Redeemer’s University, Ede 232103, Osun State, Nigeria; 5 Department of Measurement and Evaluation in Education, Recep Tayyip Erdoğan University, Campus Zihni Derin, Fener Mahallesi, 53100 Rize, Turkey; 6 Department of Psychology and Psychotherapy, Ukrainian Catholic University, Sventsitskogo 17, 79011 Lviv, Ukraine; 7 eHealth, Research Centre, C/Cristofol de Boleda, 1-2, 25006 Lleida, Spain

**Keywords:** vaccination, fear, anxiety, personality type D, alexithymia, network analysis

## Abstract

This study examines the links between vaccination status, fear of vaccination (cognitive and somatic symptoms), anxiety, alexithymia, and type D personality (negative affect and social inhibition), to propose policies to increase vaccination rates. A sample of university students (*n* = 2535; mean age = 20.59, *SD* = 2.04; male: 26.75%, female: 73.25%) from Spain, Italy, Lebanon, Nigeria, Turkey, and Ukraine completed the Vaccination Fear Scale (VFS-6), the Generalised Anxiety Disorder scale (GAD-7), the Perth Alexithymia Questionnaire—Short Form (PAQ-S), the Type D Scale (DS14), and also a question on vaccination status. Correlation, regression, and network analyses were applied. Cognitive symptoms of fear of vaccination and negative affect were the most significant in the correlation and regression analyses. In the network analysis, negative affect showed the highest values in all centrality indices and positive relationships with other nodes. Vaccination status showed negative relationships with fear of vaccination, alexithymia, and social inhibition. The network structure is similar between the sexes but varies between cultures and sexes within cultures. The relationship between vaccination status and cognitive symptoms of fear of vaccination was the most consistent, allowing for interventions at this level to be advised across cultures. For more specific interventions, cultural context must be considered for optimal results.

## 1. Introduction

There is consensus that vaccination constitutes a crucial intervention that prevents between two and three million deaths per year, standing out as one of the most economically efficient strategies for the prevention of infectious diseases [1,2]. Despite these clear benefits, vaccination rates have seen a decline in recent years, emerging as one of the top ten threats to global health [2]. Consequently, the imperative to improve vaccination rates has been captured in the strategic framework supporting the Sustainable Development Goals (SDGs) outlined in the 2030 Agenda, a commitment made by all member states of the United Nations [1].

The existing literature on vaccine hesitancy and resistance primarily focuses on describing sociodemographic factors. In this context, risk factors for vaccine resistance have been identified as being young, female [3,4], single, childless, from rural areas [5], with a low educational level [5], low income [5,6], without health insurance [3], and belonging to a minority ethnic group [5,6]. Additionally, mistrust of authorities and health experts and certain political views [7,8,9], religious or personal beliefs against vaccination [3,10], and other conspiracy beliefs [11,12,13] have also been described.

Studies on psychopathological factors or mood states are less common. Among specific phobias, trypanophobia, or fear of needles, has been described as a factor of reluctance [11,12,14]. An underlying factor in vaccine refusal is fear of vaccination [15,16,17], which has been shown to be a more significant predictor than vaccine hesitancy [18] or fear of becoming infected with the disease [16]. Therefore, as symptoms of fear are easily identifiable and interventions exist to manage them, researchers in this field suggest developing strategies to reduce fear of vaccination [19].

Fear and anxiety often show a good correlation, but there are no data to establish a positive relationship between anxiety and vaccination status [20]. Theoretically, people with anxiety disorders tend to overestimate threats [21], have a greater intolerance of uncertainty [22], and may be more hesitant than those without anxiety problems [23], which could affect their decision to get vaccinated. In any case, studies conducted during the COVID-19 pandemic, when health authorities were urging vaccination, show that anxious and non-anxious individuals did not differ in terms of COVID-19 vaccine hesitancy and acceptance [24]. There are also data indicating that COVID-19-related anxiety was positively associated with vaccination [20]. This contrasts with other studies suggesting that the fear of vaccination correlates positively with generalised anxiety [25].

It is known that personality traits exert a considerable influence on health behaviour [26]. In this context, traits which are regarded as risk factors for vaccine rejection include paranoid tendencies and low levels of conscientiousness, agreeableness, emotional stability, and empathy [8,27]. Similarly, studies indicate that individuals with lower levels of emotional stability, conscientiousness, and agreeableness, but higher levels of openness to experience, expressed less confidence in the safety of vaccines [3,5]. Openness to experience, conscientiousness, extraversion, agreeableness, and negative emotionality were associated with COVID-19 vaccine rejection; however, the facets of agreeableness, conscientiousness, and openness tended to lose importance in favour of negative emotionality and extraversion as the circumstances of the pandemic changed [28]. These results suggest a possible connection between type D personality and alexithymia with vaccine hesitancy.

There is little evidence on the impact of alexithymia or type D personality on vaccine hesitancy. Both are known to be risk factors for anxiety, depression [29,30], and various physical pathologies [29]. Alexithymia is a multidimensional personality trait characterised by difficulties in identifying and describing emotions, an impoverished fantasy life, and an outward-oriented thinking style, which could be relevant to vaccine hesitancy [31,32]. The rationale for suggesting a predictive role of alexithymia in vaccine hesitancy is based on the concept that the lack of access to personal emotions and emotional empathy compromises the ability to understand the emotional significance of protecting oneself and others from infection through vaccination [33]. Type D personality was defined by Denollet et al. [34] as a distressed personality construct characterised by the simultaneous and synergistic interplay of its two components: negative affectivity (NA), or a tendency towards negative thoughts and emotions, and social inhibition (SI), or difficulty in expressing such thoughts and emotions. SI has been associated with high negative emotionality and personal distress [35] and has a high, significant, and negative correlation with extraversion [36]. When individuals experience a life-threatening situation, negative affect (expressed as fear and anxiety) can motivate them to act in the interest of their health [37] and promote vaccination [38]. However, a recent study revealed that negative affect correlated negatively with intentions to vaccinate against COVID-19 [39], which aligns with the hypothesis that in uncertain and uncontrollable situations, individuals focus on reducing negative affect rather than reducing potential threats through behavioural changes [40]. Therefore, fostering vaccination intentions through an individual’s negative affect may stimulate more fear and the inability to adopt preventive measures such as vaccination [39,41].

In this way, the objective of this study was to investigate the relationships of potentially relevant factors such as fear of vaccination, generalised anxiety, alexithymia, type D personality (negative affect and social inhibition), related to vaccination status, taking into account the sex and culture of the study population The ultimate goal is to identify common factors, regardless of context, that influence the decision to get vaccinated, enabling the design of pro-vaccination campaigns that are applicable across different settings, making them more cost-effective and feasible in public health.

The network analysis was applied in the present study. This methodology has already been used in previous studies to investigate the association between different psychological alterations, e.g., [42,43]. This type of analysis is characterised by providing a comprehensive visual representation of the complex relationships between the variables under study, which facilitates a deeper understanding of the direct and indirect interactions between variables from a data-driven perspective. This is challenging when using more traditional multivariate techniques [42,43]. Additionally, it offers the advantage of identifying central and influential variables within the structure, bridge nodes that specifically connect the variables under study, and the strength of the interconnections [42,44]. This can help to underpin specific interventions and strategies for addressing these issues [43].

## 2. Methods

### 2.1. Participants

The sample comprised college students from Spain (*n* = 388), Italy (*n* = 376), Lebanon (*n* = 487), Nigeria (*n* = 561), Turkey (*n* = 410), and Ukraine (*n* = 313). Of the pooled data set (*n* = 2535) which included less than 25-year-old students only (*M*_age_ = 20.59, *SD* = 2.04), 73.25% were females while 26.75% were males. Table 1 shows additional background information.

The inclusion/exclusion criteria were being a university student under 25 years of age and being fluent in the language used in the forms. University students were selected to ensure homogeneous samples with respect to academic level and other social characteristics. A sample size of 250 or more per country was required because simulation studies have shown that sample sizes of 250 are generally sufficient for networks of up to 10 to 25 nodes [45,46]. In this case, the network consists of 7 nodes (1 binary and 6 continuous), so the sample size is considered appropriate for the current study.

### 2.2. Procedure

The countries included in this study (Spain, Italy, Lebanon, Nigeria, Turkey, and Ukraine) were selected through an open call for researchers interested in collaborating on the study. Those who participated voluntarily accepted the invitation, demonstrating their commitment to contributing to the research.

The data were collected in 2023, during the final phase of the COVID-19 pandemic, prior to the WHO declaration of the end of the health crisis, when a booster vaccination was recommended but not mandatory. The articles found on this topic were written before the pandemic or during its critical phases, when the perception of disease risk was high, and the importance of immunisation was significant. However, there are few studies focused on the final phase of the pandemic, when the risk perception still existed but was lower, and there was more information about the safety and effectiveness of the vaccines that would be received. Hence, this research could be timely in offering evidence-based updates on predictive vaccination variables in the absence of a pandemic emergency, which could be relevant for any vaccination programme.

The questionnaire was administered in the official language of each participating country (Spanish, Italian, Arabic, English, Turkish, and Ukrainian). The scales are already validated in these languages, so cross-cultural adaptation was not required. The questionnaire was administered online. The link to the questionnaire was sent to university professors, requesting their voluntary collaboration in the study. Those who agreed forwarded the link to their students. Students who voluntarily clicked on the link first accessed the informed consent document. This document informed potential participants about the study’s objectives, the intended use of the data, and the measures taken to ensure the confidentiality of the data collected. Explicitly providing informed consent was a necessary requirement for continuing and participating in the study. The Ethical Review Committee of the University of Lleida was responsible for granting ethical approval for this study (study authorisation number: CERT23).

### 2.3. Instruments

*Vaccination Status:* A binary question (yes/no) was included to determine whether the participant had received the vaccine.

*Vaccination Fear Scale* (VFS-6; [16,25]): This scale consists of 6 items, divided into two correlated factors with 3 items each. The first factor assesses cognitive symptoms of the fear of vaccination (F1: items 1, 2, and 4), while the second factor evaluates somatic symptoms (F2: items 3, 5, and 6). The items are rated on a 5-point Likert scale, ranging from 1 (strongly disagree) to 5 (strongly agree), with total scores ranging from 6 to 30. Higher scores indicate higher levels of fear of vaccination. The items are applicable to any vaccine; in this case, they were adapted to assess fear of the COVID-19 vaccine. In this study, the scale from Spain, Italy, Lebanon, Nigeria, Turkey, and Ukraine revealed a Cronbach’s α from 0.83 to 0.88; a cognitive factor from 0.80 to 0.89; and a somatic factor from 0.81 to 0.91.

*Generalised Anxiety Disorder* (GAD-7; [47]): This scale comprises 7 items that assess symptoms such as feeling nervous or anxious; excessive worry about various things; easy irritability; or a sense of fear that something terrible might happen. The items are scored on a 4-point Likert scale, ranging from 0 (not at all) to 3 (every day), with total scores ranging from 0 to 21. Scores above 10 are considered to be in the clinical range. In this study, Cronbach’s α values of 0.86 to 0.91 were obtained for the scales used in Spain, Italy, Lebanon, Nigeria, Turkey, and Ukraine.

*Type D Personality Scale* (DS14; [48]): The DS14 is a 14-item scale with two subscales. One subscale measure negative affectivity (NA: items 2, 4, 5, 7, 9, 12, and 13), and the other measure social inhibition (SI: items 1, 3, 6, 8, 10, 11, and 14). Items are rated on a 5-point Likert scale ranging from 0 = false to 4 = true (items 1 and 3 are reverse-scored). In this study, the Cronbach’s alpha obtained for the total sample was 0.86 and 0.80 for NA and SI, respectively. In this study, the scale from Spain, Italy, Lebanon, Nigeria, Turkey, and Ukraine revealed a Cronbach’s α from 0.83 to 0.90; an NA factor from 0.80 to 0.89; and an SI factor from 0.66 to 0.88.

*Perth Alexithymia Questionnaire—Short Form* (PAQ-S; [49]): This is a unidimensional scale consisting of 6 items, scored on a 7-point Likert scale ranging from 1 (completely disagree) to 7 (completely agree). Higher scores indicate higher levels of alexithymia. In the total sample of this study, the Cronbach’s alpha obtained was 0.83, indicating good internal consistency. In this study, the scale from Spain, Italy, Lebanon, Nigeria, Turkey, and Ukraine revealed a Cronbach’s α from 0.79 to 0.87.

### 2.4. Statistical Analysis

Descriptive, frequency, and inferential analyses were conducted using the SPSS v.28 software. The quantitative variables on a ratio scale were subjected to the Kolmogorov–Smirnov normality tests (*n* > 50). None of the variables fulfilled the assumption of normality (*p* < 0.05), so they were analysed through non-parametric inferential tests. Consequently, for the analysis of differences, medians and interquartile ranges were calculated for continuous variables, such as scores on the applied scales, while frequencies were computed for categorical variables, such as vaccination status. Subsequently, the Kruskal–Wallis test was applied to compare the medians of continuous variables and the Chi-square test to compare the frequencies of categorical variables. Spearman’s *Rho* coefficient was used to evaluate the correlations between the main variables. The strength of the association and its directionality were assessed by Gamma (*Γ*) and Somers’ *d* statistics, respectively. Finally, the predictive capacity of the variables on vaccination status was determined by hierarchical regression analyses. Due to the absence of prior studies providing a specific order of importance for the variables in our particular context, we opted to use the SPSS automatic stepwise approach. This method allows us to evaluate the incremental contribution of each variable, providing valuable information about their relative influence in the model.

Following this, using the JASP v.0.18.3.0 package, network analysis was applied to elucidate and confirm the factorial structure of the different scales used in the study and their independence (based on the items from the different applied scales). Subsequently, network analysis was conducted for the data on vaccination status, fear of vaccination measured with the VFS-6 as cognitive fear and somatic fear, generalised anxiety disorder measured with the GAD-7, alexithymia measured with the PAQ-S, and type D personality measured with the DS14 as negative affect and social inhibition.

The analysis was conducted following the recommendations of Epskamp et al. [50]. The first step involved the use of a pairwise Markov random field (PMRF; [51]). Specifically, a Mixed Graphical Model (MGM) with regularised estimation using the extended Bayesian information criterion (EBIC) was used [50]. MGM is applied as it is the most appropriate technique for processing data that contain continuous and categorical variables [52]. An EBIC is used to estimate model parameters and select the most appropriate network structure while avoiding overfitting by allowing for simpler and more parsimonious models to be chosen [50].

When used to conduct network psychometrics, in the MGM, using PMRF, the variables are represented as nodes in a graph, and connections or relationships between variables are represented as edges. These edges are grouped into ordered and related subnetworks, forming clusters similar to latent variables [53], indicating the degree to which they represent a dimension, thereby demonstrating whether the components significantly measure a construct [54]. In the second case, the analysis is applied to elucidate the network structure of the continuous and categorical variables in the study. The network plot was used to establish the conditional dependence of each node considering all other nodes in the network [55]. To obtain information about the relevance of each node, four centrality indices were estimated: betweenness, closeness, strength, and expected influence [50]. In this study, particular attention was paid to the edges between nodes, both positive and negative, and edge weights that represent the strength of the conditional association between nodes. Finally, following Hevey’s [55] recommendations, the stability of the nodes was also examined using the subset bootstrap procedure based on 1000 iterations [50].

## 3. Results

### 3.1. Descriptive, Frequency, and Inferential Analysis

The descriptive and frequency analysis are shown in Table 1. The results reveal statistically significant differences between the countries for all analysed variables (*p* < 0.001). Vaccination rates vary widely, from 37.61% in Nigeria to 96.40% in Spain. Analysis of vaccination fear (VFS-6) shows medians ranging from 8.0 in Spain and Italy to 14.0 in Nigeria. The cognitive fear component (VFS1) has medians of 5.0 in Spain and Italy, and 9.0 in Nigeria and Turkey, while the somatic fear (VFS2) shows less variability, with medians of 3.0 in most countries and 4.0 in Lebanon and Nigeria. The medians for generalised anxiety disorder (GAD-7) are relatively homogeneous, varying between 7.0 in Turkey and 10.0 in Spain. For alexithymia (PAQ-S) and type D personality, medians range from 15.0 and 34.0 in Turkey to 22.0 and 40.0 in Nigeria, respectively, reflecting differences in emotional regulation and levels of negative affect and social inhibition between the countries.

In summary, countries with high vaccination rates, such as Spain and Italy, show the lowest medians for vaccination fear. In contrast, Nigeria, with the lowest vaccination rate, has the highest medians for vaccination fear, as well as for alexithymia, negative affect, and social inhibition.

### 3.2. Correlation and Regression Analysis

As detailed in Table 2, and as expected, all analysed variables show significant Spearman Rho correlation with each other and with vaccination status, with significance levels at 0.01 (two-tailed). Among these variables, cognitive fear (VFS1) exhibits the most notable correlation with vaccination status (*Rho* = −0.311), indicating a moderate negative relationship. The importance of this relationship is further supported by the directionality and strength of the association (*Γ* = −0.454; *d* = −0.239). Following this, alexithymia (PAQ-S), cognitive fear (VFS2), and social inhibition (SI) present weaker negative correlations, with directionality and strength of association values consistent with these results. Although significant, negative affect (NA) and anxiety (GAD) show much weaker positive correlations.

The results for hierarchical regression analysis (see Table 3) indicated that vaccination status was more strongly associated with the cognitive factor of fear of vaccination (VFS1), thereby explaining the 10.3% vaccination status in the total sample, during the final phase of the pandemic. The introduction of the other variables step by step allowed for an increase in explained variance to 15.8%. Generalised anxiety did not increase the explained variance in the total sample, confirming the results obtained in the correlation analysis.

### 3.3. Psychometric Network Analysis

**Figure 1:**
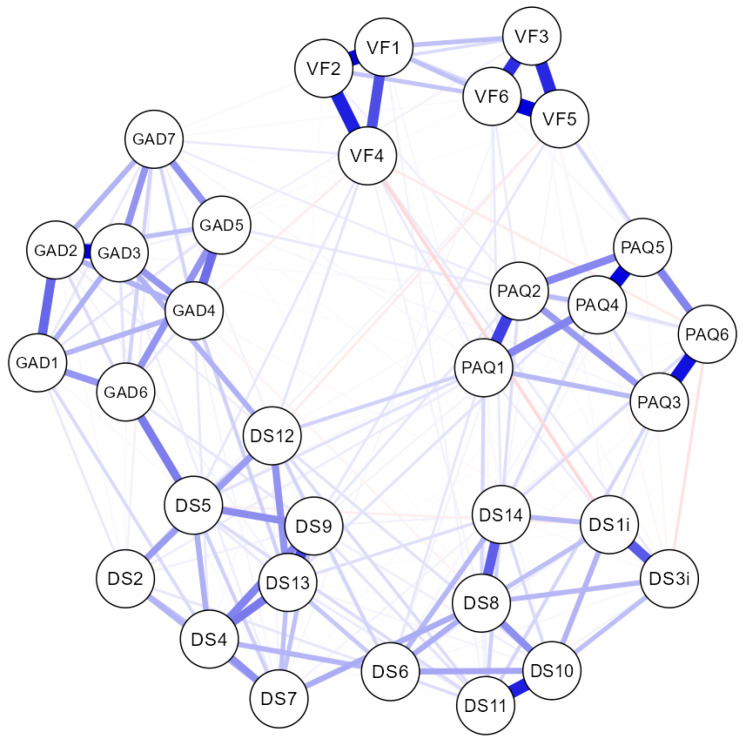
The network obtained for the total sample. VF: VFS-6 items. GAD: GAD-7 items. PAQ: PAQ-S items. DS: DS14 items.

The results of the network analysis, after introducing all the items from the questionnaires (VFS-6, GAD-7, PAQ-S, and DS16), can be seen in Figure 1. The items were clearly grouped as expected, in ordered and related subnetworks, forming clusters that corresponded to the latent variables of this study. This demonstrated that the components significantly measured their corresponding constructs, indicating good independence among them.

The network analysis, depending on sex, showed the same clusters, but with a sparsity (related nodes/total nodes) of 0.648 in males and 0.593 in females (being 0.589 in the total sample). This suggested a greater interrelation among items from different scales and subscales in females than in males. The network analysis, depending on the country, revealed the same node grouping and clusters, but with a sparsity ranging from 0.746 to 0.636. This suggested a more complex interrelation among items in the Lebanese sample, followed by the Nigerian, Ukrainian, Turkish, Spanish, and Italian samples, respectively.

Strong relationships were observed between certain items of the GAD-7 (items 3 and 6) and NA (items 5 and 12), particularly in the Ukrainian sample and to a lesser extent in the Lebanese sample. In contrast, in the Lebanese sample, the items of the DS14 formed a well-defined cluster, but not the NA and SI subnetworks, which, although ordered in proximity as expected, exhibited complex relationships among their items and also with the PAQ-S and VFS-6. The results of this analysis can be found in the Supplementary Material.

### 3.4. Network Analysis

**Figure 2:**
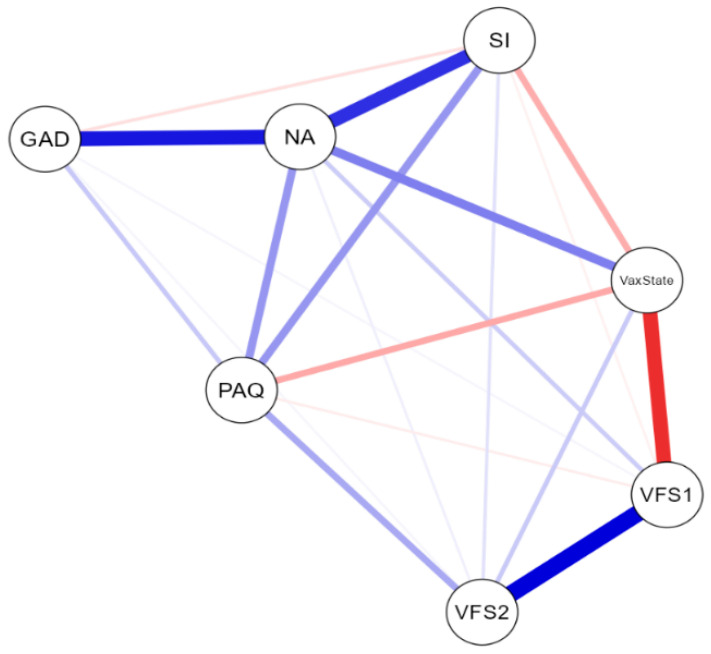
Total sample network plot (*n* = 2.535). VFS1: cognitive fear measured with VFS-6. VFS2: somatic fear measured with VFS-6. GAD: generalised anxiety disorder measured with GAD-7. PAQ: alexithymia measured with PAQ-S. NA: negative affect measured with DS14. SI: social inhibition measured with DS14.

The NwA plot, incorporating the total scores of the VFS-6, GAD-7, PAQ-S, and DS14 scales and subscales (continuous variables), as well as responses to the dichotomous vaccination status question (categorical variable), is shown in Figure 2. The network displayed seven nodes, with a sparsity of 0.048, indicating a clear interrelation among all the nodes.

**Figure 3:**
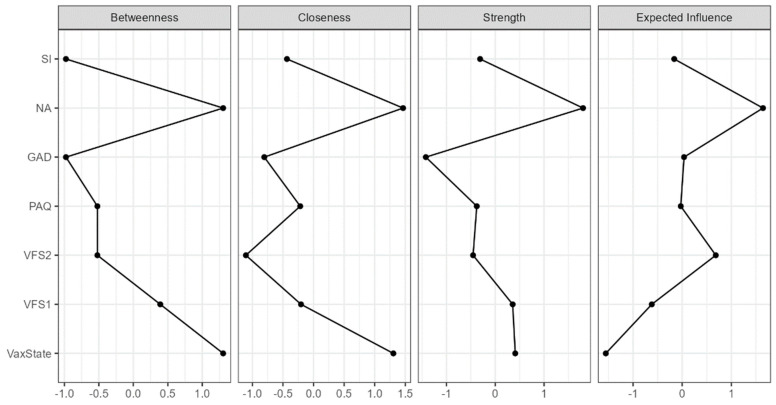
Centrality plots for total sample network analysis (*n* = 2535). VFS1: cognitive fear measured with VFS-6. VFS2: somatic fear measured with VFS-6. GAD: generalised anxiety disorder measured with GAD-7. PAQ: alexithymia measured with GAD-7. NA: negative affect measured with DS14. SI: social inhibition measured with DS14.

The network weight matrix and centrality measures for the total sample can be seen in Table 4. Centrality plots are available in Figure 3. The node showing the highest centrality in the network was negative affect, followed by vaccination status. Negative affect was positively connected with vaccination status (*r* = 0.251; *p* < 0.05), anxiety (*r* = 0.438; *p* < 0.05), and all other nodes in the network.

On the other hand, vaccination status, besides exhibiting a strong positive relationship with negative affect, showed negative edges with the cognitive factor of vaccination fear (*r* = −0.398; *p* < 0.05), social inhibition (*r* = −0.163; *p* < 0.05), and alexithymia (*r* = −0.172; *p* < 0.05).

Anxiety lacked centrality, but it exhibited positive values of expected influence in the network. Similarly, the somatic factor of vaccination fear did not show adequate centrality values, but it could exert its effect through the cognitive factor, which displayed positive values of strength and betweenness centrality, explaining the weight matrix between factors (VFS2/VFS1: *r* = 0.493; *p* < 0.05; VFS2/VS: *r* = 0.105; *p* < 0.05).

**Figure 4:**
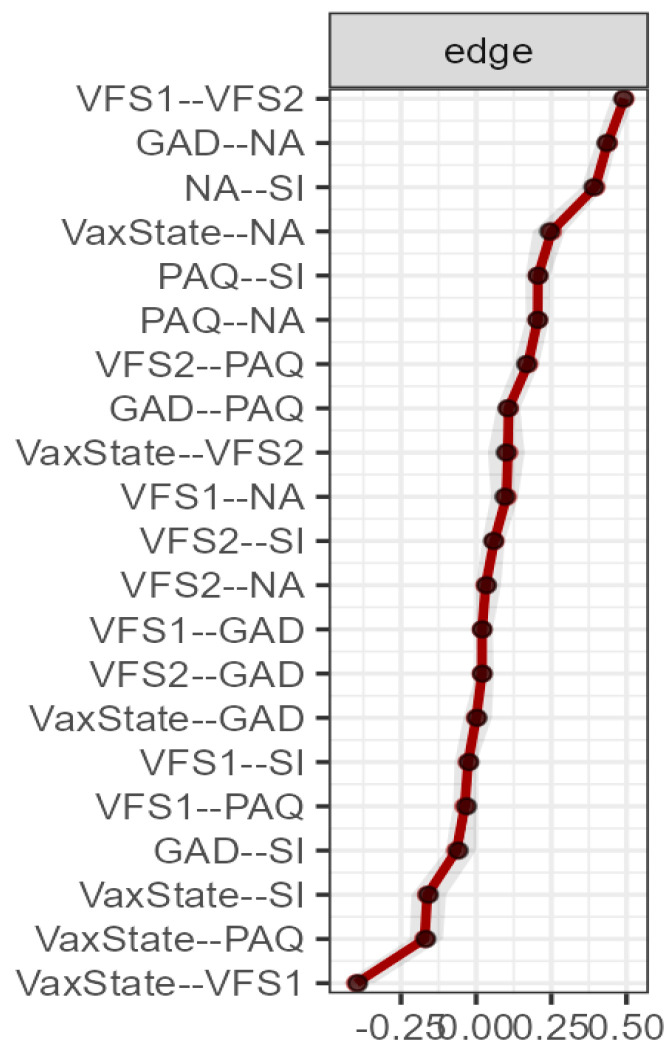
Edge stability for total sample network analysis (*n* = 2535). The edge weights, with each horizontal line representing an edge, are represented by the red line, and the 95% confidence intervals by the grey area. VS: vaccination state. VFS1: cognitive fear measured with VFS-6. VFS2: somatic fear measured with VFS-6. GAD: generalised anxiety disorder measured with GAD-7. PAQ: alexithymia measured with GAD-7. NA: negative affect measured with DS14. SI: social inhibition measured with DS14.

The stability data of the nodes, after applying a subset bootstrap procedure based on 1000 iterations, were very adequate (see Figure 4), confirming the adequacy of the sample size.

### 3.5. Network Analysis for Sex and Cultures

The network analysis depending on sex resulted in an equivalent network structure between the male and female samples and with the total sample. However, there was a lower sparsity value in females compared to males (0.143/0.238), indicating a greater number of relationships between items among females. On the contrary, the network analysis depending on culture resulted in network structures with significant differences between them and with the total sample. The results of this analysis can be found in the Supplementary Material.

The sparsity values ranged from 0.238 in Spain, Italy, and Lebanon to 0.381 in the Turkish sample, 0.429 in the Nigerian sample, and 0.476 in the Ukrainian sample (from more to fewer relationships between items). Similarly, to the total sample, negative affect exhibits the highest centrality in all samples, except for the Spanish sample, where it only appeared in the female sample. On the other hand, vaccination status retained some of its indices only in certain samples. Anxiety showed good, expected influence in all samples, regardless of sex, with notable centrality observed for all its indices in the Spanish sample of both sexes, and for some of its indices in the Nigerian, Turkish, and Ukrainian samples. Additionally, notable centrality was observed for alexithymia in the Lebanese sample and social inhibition in the Turkish sample. In general, the factors of vaccination fear became important, as they exhibit positive values in some of their centrality indices across all samples. The edge analysis revealed the presence of positive edges of negative affect with all or some of the other nodes in the network, but with a less significant weight matrix in its relationship with vaccination status. On the other hand, vaccination status showed negative edges with cognitive fear in the Nigerian, Turkish, and Ukrainian samples of both sexes and in the Italian and Lebanese samples of females. It also exhibited negative edges with somatic fear of vaccination in the Spanish male sample. Negative edges were also observed with anxiety in the Spanish female sample and with alexithymia in the Lebanese female sample and the Ukrainian male sample.

## 4. Discussion

The study involved researchers from Spain, Italy, Lebanon, Nigeria, Turkey, and Ukraine. Although the sample was not intentionally selected, it provides valuable geographical and cultural diversity, enriching the analysis of how factors such as vaccine fear, generalised anxiety, alexithymia, and type D personality influence vaccination decisions. The most significant differences pertain to the individualistic or collectivist characteristics of their societies, trust in health authorities, vaccine accessibility, and the type of vaccine administered. Spain and Italy, with an individualistic orientation, focus on personal decision-making [56]. In contrast, Lebanon and Nigeria exhibit more collectivist traits, where health decisions are often based on social norms and community expectations, as well as the opinions of community, family, or religious leaders [57,58,59,60,61,62,63]. Turkey and Ukraine, on the other hand, have mixed characteristics [56], leading to a combination of individual and collective influences on vaccination. Trust in health authorities also varies significantly among these countries, particularly in the context of the COVID-19 pandemic. In Spain, Italy, and Turkey, trust in health authorities was relatively high; in contrast, trust was lower in Lebanon and Nigeria, with misinformation significantly impacting vaccine acceptance [58,59]. In Ukraine, before the conflict, there was generally a positive disposition towards vaccines, though trust in authorities could be variable depending on the local context [59]. Vaccine accessibility also varied. In Ukraine, access to vaccines was relatively universal before the conflict [58,59], but this changed afterwards. In Nigeria, vaccine accessibility was more uneven due to challenges in health infrastructure and distribution [60]. In Lebanon, universities played a crucial role in providing vaccines to students, thereby improving accessibility for this demographic group [61]. Regarding specific vaccines administered, Pfizer-BioNTech was used in Spain, Italy, Turkey, Lebanon, and Ukraine; Moderna in Spain and Italy; AstraZeneca in Spain, Italy, Turkey, Lebanon, and Nigeria; Janssen in Spain, Italy, and Nigeria; Sinovac (CoronaVac) in Turkey and Ukraine; Sinopharm in Nigeria; and Sputnik V in Turkey and Ukraine [59,60,61]. Generally, Pfizer-BioNTech, Moderna, and AstraZeneca encountered less rejection compared to vaccines such as Sinovac, Sinopharm, and Sputnik V [62].

The results of the correlation analysis showed a significant and negative relationship between cognitive fear of vaccination and vaccination status. Directionality and strength results support this finding, indicating it as a factor to consider. Correlations were also negative, although of low magnitude, with alexithymia and social inhibition. These findings suggest that, although there is a relationship, it is relatively weak, indicating that these factors have limited influence on the decision to vaccinate. Contrary to expectations, negative affect showed a positive correlation, albeit of low magnitude. Therefore, its effect would have a very limited impact on vaccination status. Moreover, although the initial hypothesis suggested that generalised anxiety might be related to vaccination status, the results did not show a significant correlation between these variables. This finding could suggest that generalised anxiety is not a relevant predictor of vaccination behaviour in this specific sample. The regression analysis confirmed the results of the correlation analysis for cognitive symptoms of vaccination fear, indicating an explanatory capacity of 10.3%. However, the regression also showed that negative affect, despite its positive correlation, increases the explained variance of the model from 10.3% to 12%, suggesting an additional significant predictive effect. Similarly, social inhibition and alexithymia increase the explained variance up to 15.8%, reflecting their predictive importance.

The network analysis can provide an explanation for these results. This analysis allowed for the identification of central variables and the most influential interactions within the network structure generated by the study variables. This approach provided a deeper understanding of how the studied factors interrelate, highlighting negative affect as a central node that connects all variables to each other. Thus, while it presented a positive edge with vaccination status, it also exhibited edges with anxiety, alexithymia, social inhibition, and the vaccination fear factors. Therefore, although it has a direct positive effect, indirectly it could have a negative effect, explaining its capacity to increase the explained variance in vaccination status.

The results of this study suggested that vaccination fear, especially the cognitive fear factor, was significantly related to vaccination status. This finding is consistent with previous research that identifies fear as a crucial factor in vaccine hesitancy [15,16,17], with fear of vaccination being a stronger predictor compared to hesitancy [18] or fear of the targeted disease [16]. The negative association between cognitive fear and vaccination status indicated that those with higher levels of cognitive fear are less likely to get vaccinated, underscoring the need for specific interventions to reduce this type of fear, in particular, because fear symptoms were relatively identifiable and manageable [19].

Contrary to the hypothesis proposed, generalised anxiety did not show a significant correlation with vaccination status. This finding aligns with studies conducted during the COVID-19 pandemic, where no significant differences in vaccine hesitancy were found between anxious and non-anxious individuals [24]. Thus, while some studies have found positive correlations between anxiety and vaccination fear [16,25], others suggested that anxiety about COVID-19 may be positively associated with vaccination [20]. The lack of a significant correlation could indicate that generalised anxiety is not a reliable predictor of the intention to vaccinate in the context of the final phase of the pandemic. This may also be because its variability overlapped with that of other variables, not providing additional relevant information to explain the variability of the dependent variable. In any case, the network analysis indicated that, although anxiety lacks centrality, it exhibited positive values of expected influence in the network, suggesting that it may exert its effect through other variables.

The results also suggested that difficulty in identifying and expressing emotions (a characteristic of alexithymia) and a tendency to avoid social interaction (typical of type D personality) may contribute to vaccine hesitancy. These findings are consistent with studies that reported that a lack of access to personal emotions and emotional empathy can compromise the ability to understand the importance of vaccination [33]. Additionally, although negative affect showed positive relationships with vaccination status, it also exhibited them with alexithymia and social inhibition. Denollet [48] described how in type D personality, the combination of negative affect and social inhibition can result in avoidance behaviours. However, in the context of vaccination, the NA component alone can drive preventive behaviour, which would be consistent with the hypothesis of Ferrer and Klein [56], who suggested that individuals with a high perception of risk are more likely to adopt preventive behaviours. In the current study, it is observed that negative affect without the presence of high social inhibition could motivate vaccination, which is consistent with the finding of a positive correlation between negative affect and vaccination.

The descriptive analysis revealed significant differences among samples from different countries in terms of sex, vaccination fear, anxiety, alexithymia, and type D personality. On the other hand, network analysis provided a detailed insight into how variables interrelate depending on sex and cultural context. Thus, no significant differences were observed between sexes, but differences were found between cultures. In all analysed countries, vaccination status showed a significant negative edge with the cognitive fear factor of vaccination. This indicated consistency in how vaccination fear negatively influences vaccination status across diverse cultures. Negative affect also emerged as a central node (in all samples except among Spanish males) thereby underscoring its importance in the network of analysed variables. Additionally, specific nodes and edges highlighted the importance of anxiety in the Spanish, Turkish, and Ukrainian samples; alexithymia in the Italian, Lebanese, Nigerian, and Ukrainian samples; and social inhibition in the Turkish and Ukrainian samples. These differences underscored the importance of cultural and sociodemographic factors when designing strategies to increase vaccination rates.

In any case, the most significant finding of our study is that, regardless of national characteristics such as collectivism versus individualism, trust in the healthcare system, or vaccine availability, fear of vaccination emerges as the most central factor in the decision to get vaccinated. This result could be extrapolated to other countries, suggesting that, regardless of context, fear of vaccination has a significant influence on vaccine acceptance. Therefore, countries facing similar challenges with vaccine acceptance could use these data to focus their communication and education strategies on addressing vaccine-related fears.

### Limitations and Conclusions

There were several limitations that need to be addressed. Firstly, the research findings can only be generalised to university students under the age of 25. It is unclear whether the results would also be applicable to the general population. Second, the non-directed selection of participating countries also presents limitations. The sample is not representative of all regions of the world, which may restrict the generalisability of the results to other geographic areas not included in the study. Therefore, it is recommended that future research consider a more strategic selection of countries to validate and expand these findings. Third, while fear of vaccination has been identified as a central factor in vaccine acceptance, the results were obtained specifically within the context of COVID-19 vaccination. Therefore, to determine whether these findings are applicable to other vaccines, additional research in those specific contexts is needed.

Another limitation of the study is that the questions about vaccination were designed with binary response options (yes/no), which did not capture variations in the reception of one or multiple doses of the vaccine or differences in vaccination schedules between countries. This simplified approach allowed for efficient and uniform data collection among participants, facilitating general comparisons of vaccine acceptance. However, it may have overlooked important factors influencing vaccination perception and acceptance, such as the number of doses received or variations in vaccination schedules. Additionally, the study was conducted during the final phase of the pandemic, when attitudes towards vaccination might have changed compared to the more critical phases of the health crisis. Therefore, it is possible that these aspects influenced the results, and a more detailed exploration of these variables in future research could provide a more comprehensive understanding of vaccine acceptance.

In any case, this study underscores the importance of fear, especially cognitive fear, in vaccine hesitancy. While generalised anxiety does not appear to be a significant predictor of vaccination intention, alexithymia and type D personality do, albeit to a lesser extent. Additionally, cultural and sociodemographic differences highlight the need for personalised approaches to increase vaccination rates. Network analysis emerges as a valuable tool for understanding the complex interactions among factors influencing vaccination, providing a solid foundation for future public health interventions and strategies.

## Figures and Tables

**Table 1:** Descriptive and frequency analysis (TS: total sample, *n* = 2535; 1. Spain, *n* = 388; 2. Italy, *n* = 376; 3. Lebanon, *n* = 487; 4. Nigeria, *n* = 561; 5. Turkey, *n* = 410; and 6. Ukraine, *n* = 313).

		Sample Nationality							*X^2^*/df
Variable		TS	1	2	3	4	5	6	*p*
Vaccination (%)	Yes	76.41	96.40	96.01	87.47	37.61	92.93	58.79	707.27
	No	23.59	3.60	3.99	12.53	62.39	7.07	41.21	<0.001
VFS-6	Median	11.0	8.0	8.0	11.0	14.0	13.0	9.0	66.78
	IR	8.0–15.0	7.0–12.0	7.0–11.0	8.0–16.0	10.0–18.0	9.0–17.0	7.0–13.0	<0.001
VFS1	Median	7.0	5.0	5.0	7.0	9.0	9.0	6.0	62.54
	IR	4.0–10.0	4.0–8.0	4.0–7.0	5.0–9.0	6.0–12.0	5.0–11.0	4.0–9.0	<0.001
VFS2	Median	3.0	3.0	3.0	4.0	4.0	3.0	3.0	54.76
	IR	3.0–5.0	3.0–3.0	3.0–3.0	3.0–7.0	3.0–8.0	3.0–6.0	3.0–4.0	<0.001
GAD-7	Median	9.0	10.0	8.0	9.0	9.0	9.0	7.0	10.29
	IR	5.0–13.0	6.0–13.0	6.0–12.0	6.0–14.0	5.0–14.0	6.0–14.0	4.0–11.0	<0.001
PAQ-S	Median	18.0	16.5	16.0	21.0	22.0	15.0	17.0	47.71
	IR	13.0–24.0	11.0–22.0	12.0–21.0	16.0–27.0	16.0–28.0	10.0–21.2	12.0–22.0	<0.001
DS14	Median	37,0	35.0	38.0	35.0	40.0	38.0	34.0	17.26
	IR	30.0–44.0	28.0–41.0	30.0–47.0	30.0–43.0	33.0–46.5	30.7–45.0	27.0–42.0	<0.001
NA	Median	18.0	17.0	20.0	18.0	18.0	20.0	16.0	14.37
	IR	14.0–23.0	13.0–21.0	16.0–24.0	15.0–23.0	13.5–22.5	15.0–25.0	13.0–21.0	<0.001
SI	Median	18.0	18.0	18.5	17.0	22.0	18.0	18.0	39.17
	IR	15.0–23.0	14.0–20.7	14.0–24.0	15.0–21.0	18.0–25.0	13.7–22.0	13.0–22.0	<0.001

VFS-6: Vaccination Fear Scale. VFS1: cognitive fear measured with VFS-6. VFS2: somatic fear measured with VFS-6. GAD-7: generalised anxiety disorder. PAQ-S: alexithymia. DS14: type D personality. NA: negative affect measured with DS14. SI: social inhibition measured with DS14. IR = interquartile range; *X^2^*/df = Kruskal–Wallis test expressed as Chi-square/df.

**Table 2:** Correlations, strength, and directionality between the analysed variables and vaccination state.

	Spearman Rho Correlations									Strength & Directionality		
	VS	VFS	VFS1	VFS2	GAD	PAQ	DS	NA	SI	*p*	*Γ*	*d*
VS	--									--	--	--
VFS	−0.280	--								--	--	--
VFS1	−0.311	0.953	--							<0.001	−0.454	−0.239
VFS2	−0.124	0.748	0.547	--						<0.001	−0.223	−0.109
GAD	0.021	0.165	0.133	0.181	--					0.285	0.031	0.016
PAQ	−0.142	0.263	0.212	0.283	0.315	--				<0.001	−0.199	−0.105
DS	−0.031	0.244	0.216	0.211	0.447	0.471	--			--	--	--
NA	0.069	0.217	0.189	0.187	0.533	0.419	0.872	--		<0.001	0.098	0.051
SI	−0.122	0.209	0.183	0.192	0.230	0.406	0.848	0.497	--	<0.001	−0.174	−0.091

Note: All correlations were significant at the 0.01 level (two-tailed) VS: vaccination state. VFS: fear measured with VFS-6. VFS1: cognitive fear measured with VFS-6. VFS2: somatic fear measured with VFS-6. DS: type D personality measured with DS14. NA: negative affect measured with DS14. SI: social inhibition measured with DS14.

**Table 3:** Hierarchical regression analysis for vaccination state versus analysed variables.

Model		*B*	*B* IC95%		*t*	*p*	*r* ^2^	Δ*r*^2^
1	VFS1	−0.321	−0.043	−0.034	−17.036	<0.001	0.103	0.103
2	VFS1	−0.344	−0.046	−0.037	−18.170	<0.001	0.120	0.017
	NA	0.133	0.006	0.012	6.996	<0.001		
3	VFS1	−0.330	−0.044	−0.035	−17.541	<0.001	0.139	0.020
	NA	0.212	0.012	0.017	9.879	<0.001		
	SI	−0.163	−0.015	−0.009	−7.577	<0.001		
4	VFS1	−0.316	−0.042	−0.033	−16.763	<0.001	0.151	0.011
	NA	0.247	0.014	0.020	11.135	<0.001		
	SI	−0.131	−0.013	−0.006	−5.930	<0.001		
	PAQ	−0.124	−0.009	−0.004	−5.839	<0.001		
5	VFS1	−0.367	−0.049	−0.039	−16.893	<0.001	0.158	0.007
	NA	0.239	0.013	0.019	10.810	<0.001		
	SI	−0.136	−0.013	−0.007	−6.173	<0.001		
	PAQ	−0.140	−0.010	−0.005	−6.557	<0.001		
	VFS2	0.104	0.009	0.023	4.643	<0.001		

VFS1: cognitive fear measured with VFS-6. VFS2: somatic fear measured with VFS-6. PAQ: alexithymia measured with PAQ-S. NA: negative affect measured with DS14. SI: social inhibition measured with DS14.

**Table 4:** Network weight matrix and centrality measures for total sample (*n* = 2535).

	Weight Matrix							Centrality Measures			
Variable	VS	VFS1	VFS2	GAD	PAQ	NA	SI	Betweenness	Closeness	Strength	Expected Influence
VS	0.000							1.303	1.305	0.410	−1.555
VFS1	−0.398	0.000						0.391	−0.205	0.357	−0.619
VFS2	0.105	0.493	0.000					−0.521	−1.106	−0.454	0.683
GAD	0.000	0.020	0.019	0.000				−0.977	−0.805	−1.422	0.038
PAQ	−0.172	−0.038	0.172	0.108	0.000			−0.521	−0.218	−0.379	−0.029
NA	0.251	0.101	0.032	0.438	0.205	0.000		1.303	1.464	1.799	1.644
SI	−0.163	−0.027	0.060	−0.065	0.206	0.396	0.000	−0.977	−0.435	−0.310	−0.163

VS: vaccination State. VFS1: cognitive fear measured with VFS-6. VFS2: somatic fear measured with VFS-6. GAD: generalised anxiety disorder measured with GAD-7. PAQ: alexithymia measured with GAD-7. NA: negative affect measured with DS14. SI: social inhibition measured with DS14.

## Data Availability

Data will be made available upon request.

## Supplementary Materials

Network analysis supporting information can be downloaded at https://www.mdpi.com/article/10.3390/bs14090761/s1.
