# Supplementary material for: Links between Vaccination Fear-, Anxiety-, Alexithymia-, and Type D Personality-Related Vaccination Decisions: A Network Analysis in a Multicultural Sample
Source: Behav Sci (Basel). 2024 Aug 29;14(9):761. doi: 10.3390/bs14090761 (PMC11428217; doi:10.3390/bs14090761)

1. Psychometrics for escales and subescales

Note that: VF: VFS-6 items. GAD: GAD-7 items. PAQ: PAQ-S items. DS: DS14 items

1.1 Network Analysis for total sample (*n* = 2.535)

Summary of Network

Number of nodes	Number of non-zero edges	Sparsity
33	217 / 528	0.589

Network plot

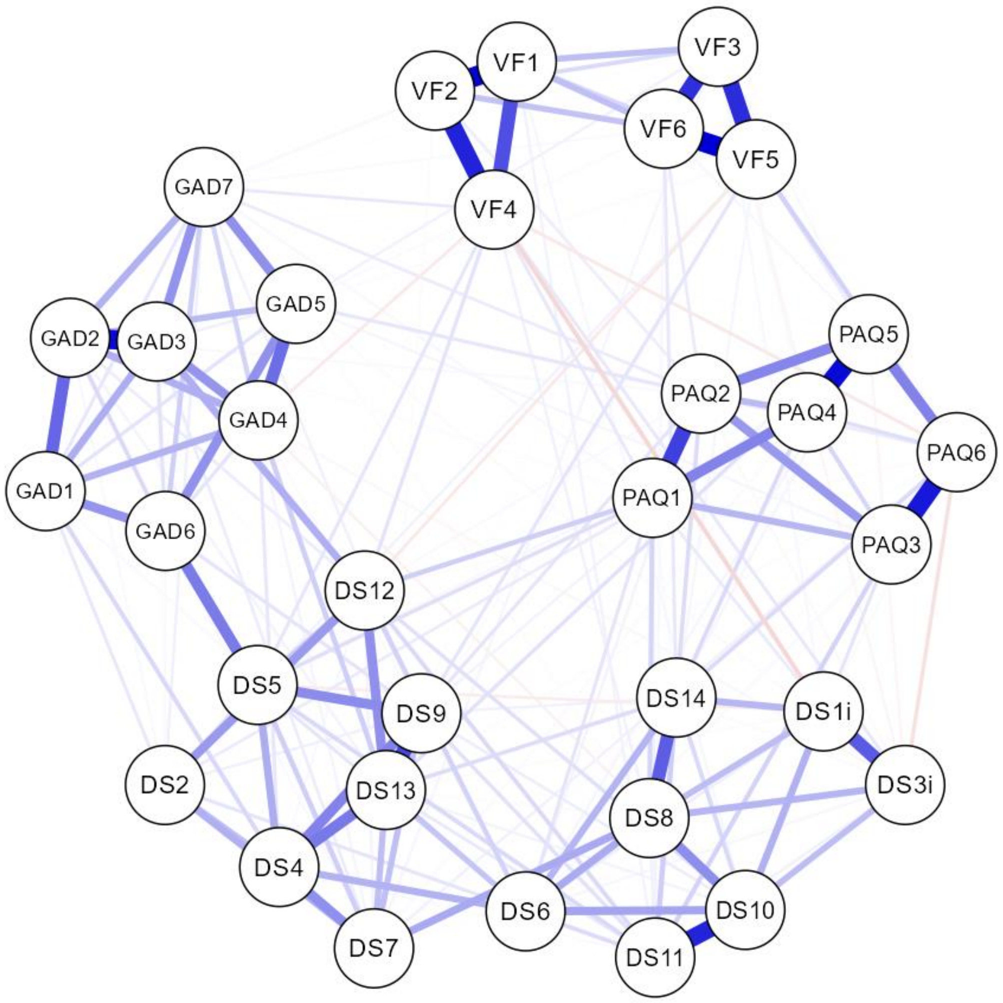

1.2 Network Analysis for male/female in total sample

Network	Number of nodes	Number of non-zero edges	Sparsity
1.Male	33	186 / 528	0.648
2.Female	33	215 / 528	0.593

1.Male network plot

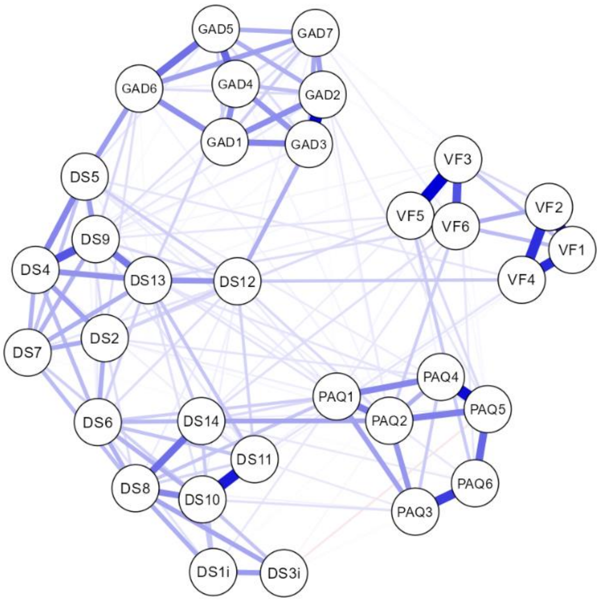

2.Female network plot

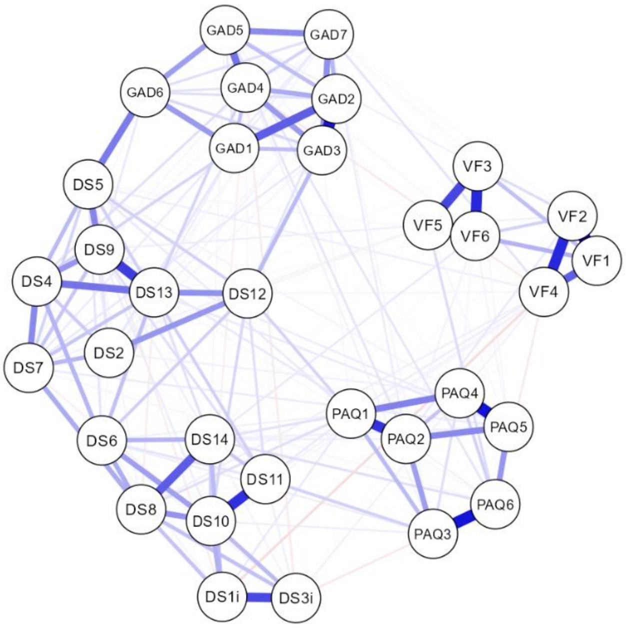

1.3. Network Analysis for the different countries under análisis

Summary of Network

Network	Number of nodes	Number of non-zero edges	Sparsity
1.Spain	33	149 / 528	0.718
2.Italy	33	134 / 528	0.746
3.Lebanon	33	192 / 528	0.636
4.Nigeria	33	186 / 528	0.648
5.Turkey	33	152 / 528	0.712
6.Ukraine	33	166 / 528	0.686

1.Spain

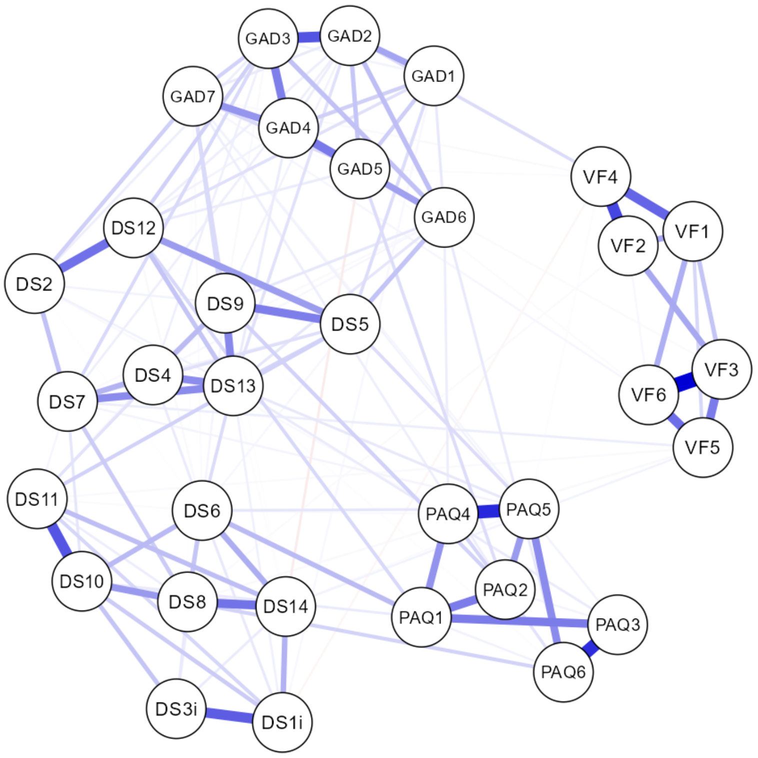

2.Italy

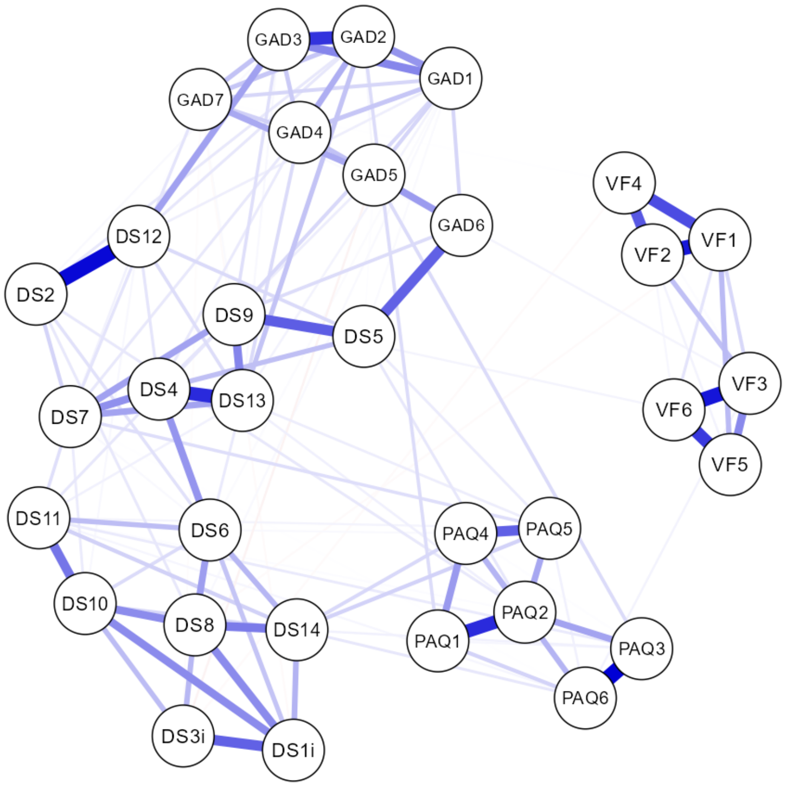

3.Lebanon

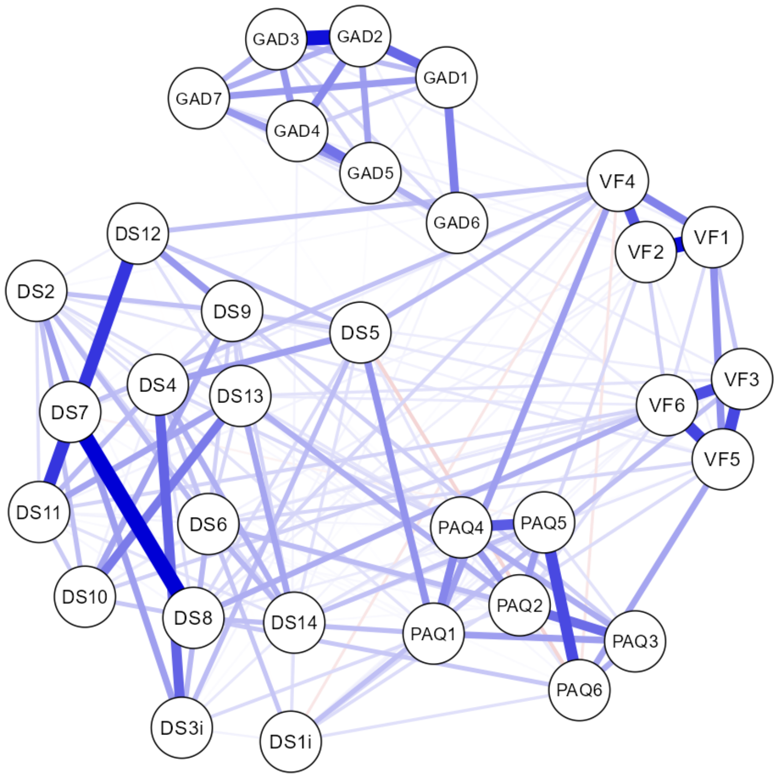

4.Nigeria

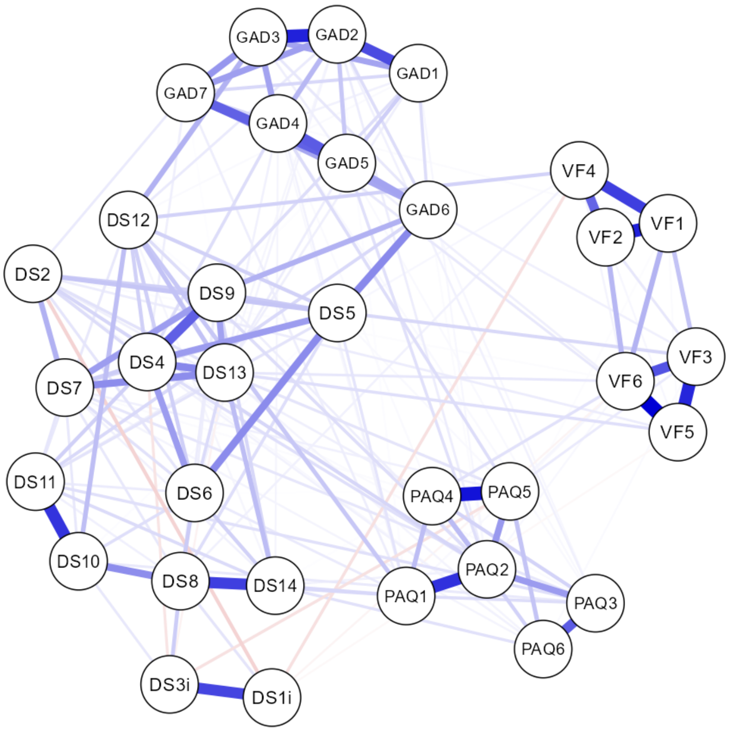

5.Turkey

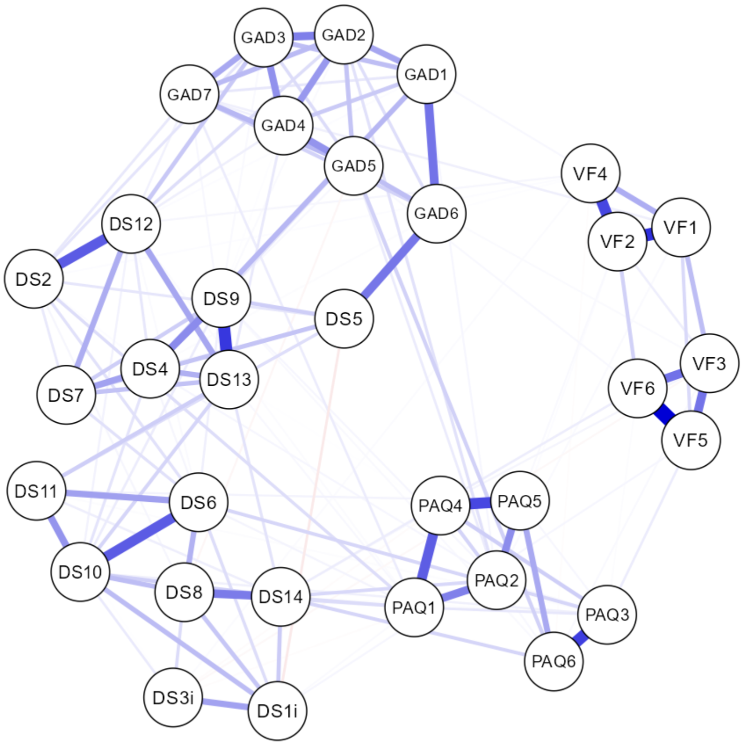

6.Ukraine

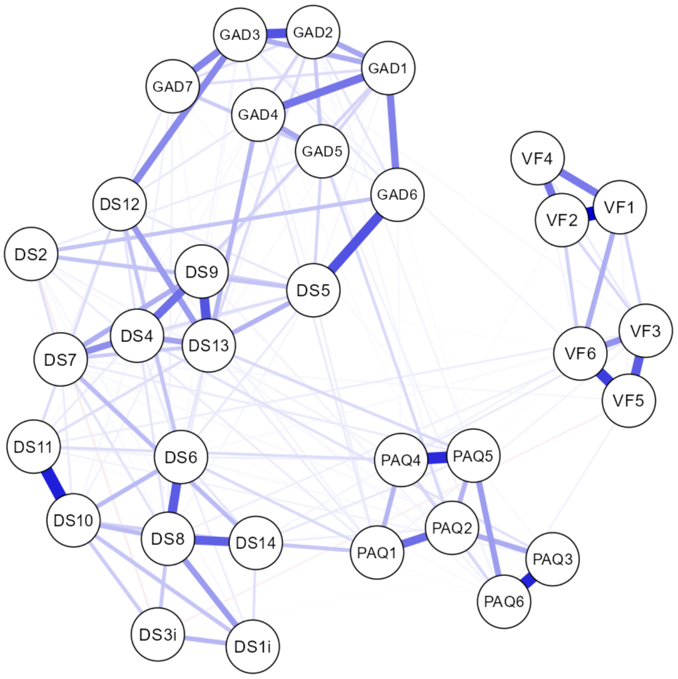
GAD3
GAD2
GAD1
GAD7
GAD4
GAD5
GAD6
VF4
VF2
VF1
DS12
DS2
DS9
DS5
DS4
DS13
DS7
VF3
VF6
VF5
DS11
DS6
PAQ4
PAQ5
DS10
DS8
DS14
PAQ1
PAQ2
PAQ3
PAQ6
DS3i
DS1i

2. Network Analysis for variables under study

Note that: VFS1: Cognitive fear measured with VFS-6. VFS2: Somatic fear measured with VFS-6. GAD: Generalised anxiety disorder measured with GAD-7. PAQ: Alexithymia measured with PAQ-S. NA: Negative affect measured with DS14. SI: Social inhibition measured with DS14.

2.1. Network análisis for total sample (*n*= 2.535)

Summary of Network

Number of nodes	Number of non-zero edges	Sparsity
7	20 / 21	0.048

Centrality measures per variable

	Network			
Variable	Betweenness	Closeness	Strength	Expected influence
VaxState	1.303	1.305	0.410	-1.555
VFS1	0.391	-0.205	0.357	-0.619
VFS2	-0.521	-1.106	-0.454	0.683
GAD	-0.977	-0.805	-1.422	0.038
PAQ	-0.521	-0.218	-0.379	-0.029
NA	1.303	1.464	1.799	1.644
SI	-0.977	-0.435	-0.310	-0.163

Weights matrix

	Network						
Variable	VaxState	VFS1	VFS2	GAD	PAQ	NA	SI
VaxState	0.000	-0.398	0.105	0.000	-0.172	0.251	-0.163
VFS1	-0.398	0.000	0.493	0.020	-0.038	0.101	-0.027
VFS2	0.105	0.493	0.000	0.019	0.172	0.032	0.060
GAD	0.000	0.020	0.019	0.000	0.108	0.438	-0.065
PAQ	-0.172	-0.038	0.172	0.108	0.000	0.205	0.206
NA	0.251	0.101	0.032	0.438	0.205	0.000	0.396
SI	-0.163	-0.027	0.060	-0.065	0.206	0.396	0.000

Total sample network plot

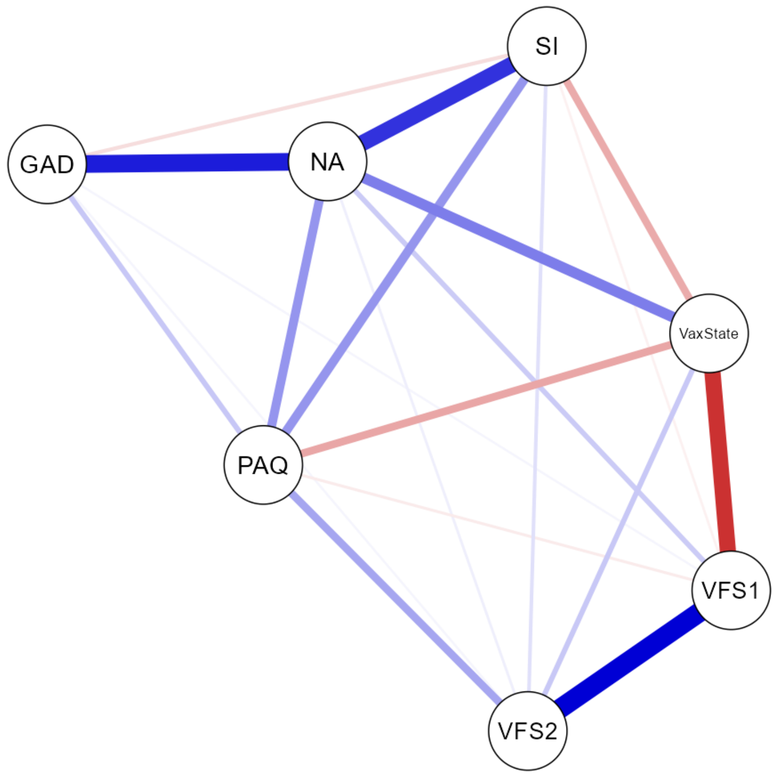

Centrality Plot

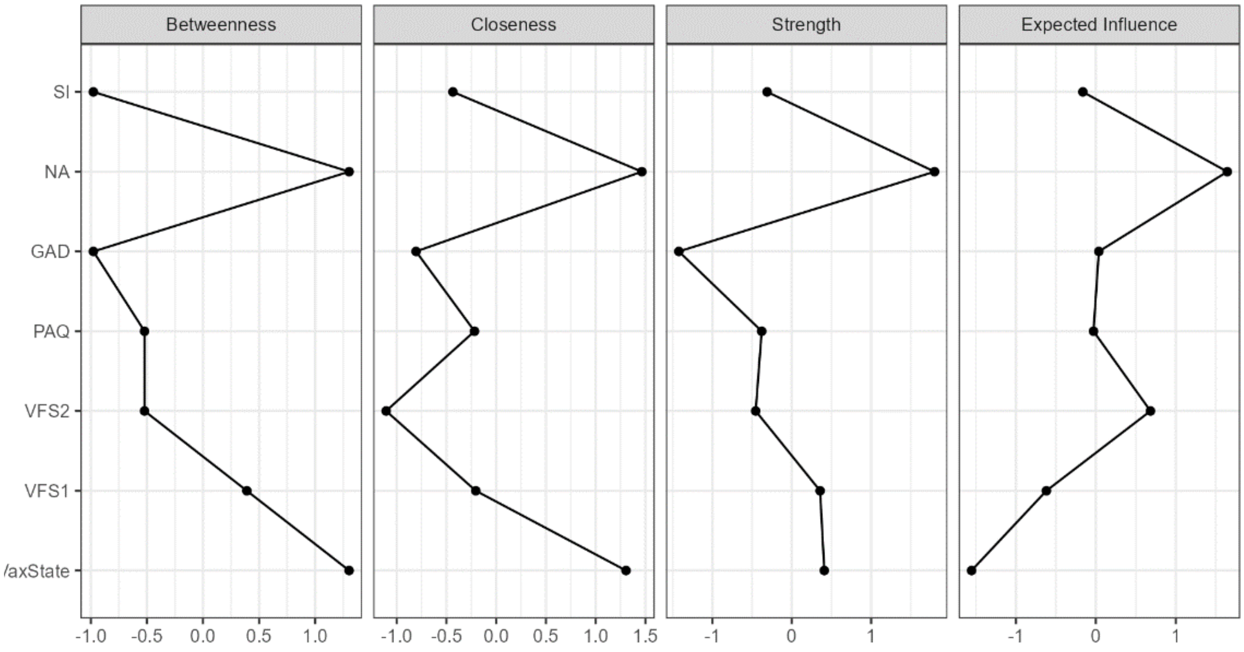

Edge Stability

Bootstrap summary of Network

Type	Number of bootstraps
Nonparametric	1000

Network

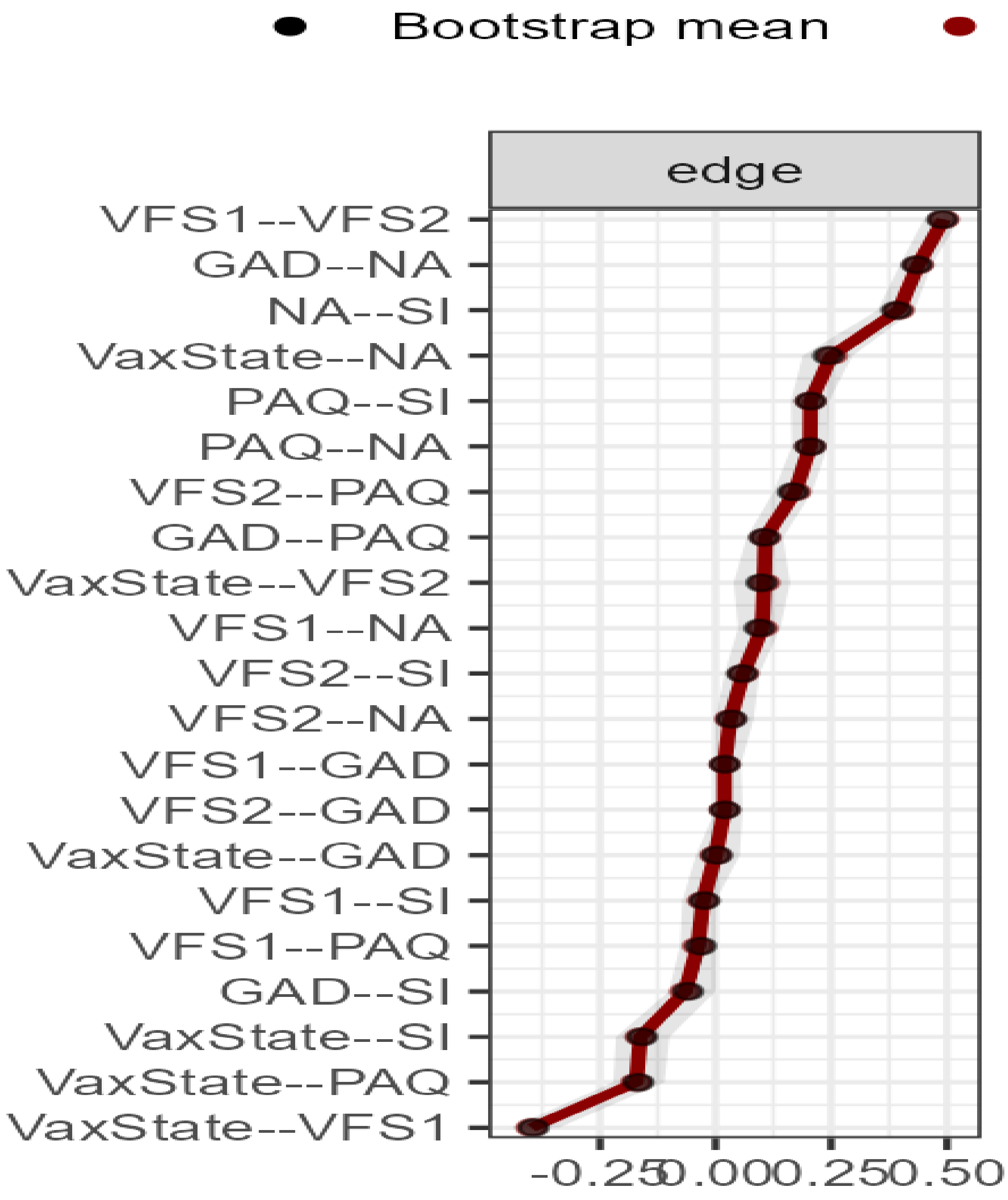

Supplementary material

Article: Links Between Vaccination Fear, Anxiety, Alexithymia and Type D Personality Related Vaccination Decision: A Network Analysis in a Multicultural Sample

Centrality Stability

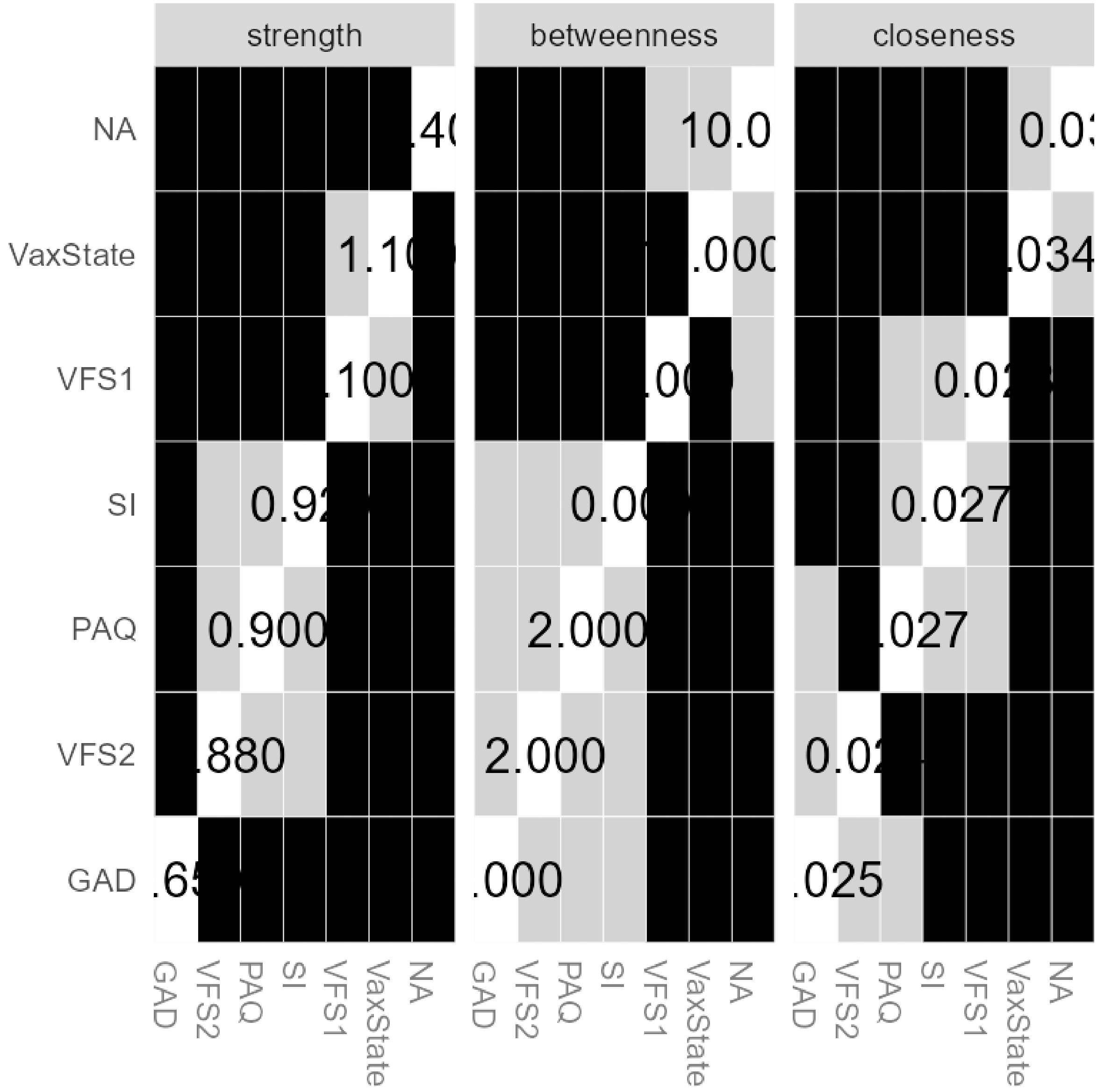

2.2. Network analysis for men/woman in total sample

Summary of Network

Network	Number of nodes	Number of non-zero edges	Sparsity
1.Male	7	16 / 21	0.238
2.Female	7	18 / 21	0.143

Centrality measures per variable

	1.Male				2.Female			
Variable	Betweenness	Closeness	Strength	Expected influence	Betweenness	Closeness	Strength	Expected influence
VaxState	0.254	0.460	-0.089	-1.577	1.193	1.215	0.386	-1.660
VFS1	-0.457	-0.300	-0.149	-0.670	0.358	-0.245	0.330	-0.566
VFS2	-0.457	-0.785	0.034	0.630	-0.756	-1.177	-0.410	0.621
GAD	-0.813	-1.094	-1.368	-0.080	-0.756	-0.576	-1.250	0.108
PAQ	0.254	0.078	-0.174	0.122	-0.756	-0.316	-0.447	0.007
NA	2.032	1.946	2.000	1.626	1.471	1.561	1.888	1.586
SI	-0.813	-0.305	-0.256	-0.051	-0.756	-0.462	-0.497	-0.096

Weights matrix

	1.Male							2.Female						
Variable	VaxState	VFS1	VFS2	GAD	PAQ	NA	SI	VaxState	VFS1	VFS2	GAD	PAQ	NA	SI
VaxState	0.000							0.000						
VFS1	-0.321	0.000						-0.389	0.000					
VFS2	0.022	0.384	0.000					0.091	0.509	0.000				
GAD	0.000	0.000	0.049	0.000				0.000	0.020	0.015	0.000			
PAQ	-0.107	0.000	0.244	0.066	0.000			-0.156	0.000	0.120	0.114	0.000		
NA	0.228	0.100	0.094	0.361	0.205	0.000		0.215	0.055	0.041	0.438	0.200	0.000	
SI	-0.142	0.000	0.061	0.000	0.175	0.397	0.000	-0.135	0.000	0.036	-0.042	0.215	0.365	0.000

Network Plots

1.Male

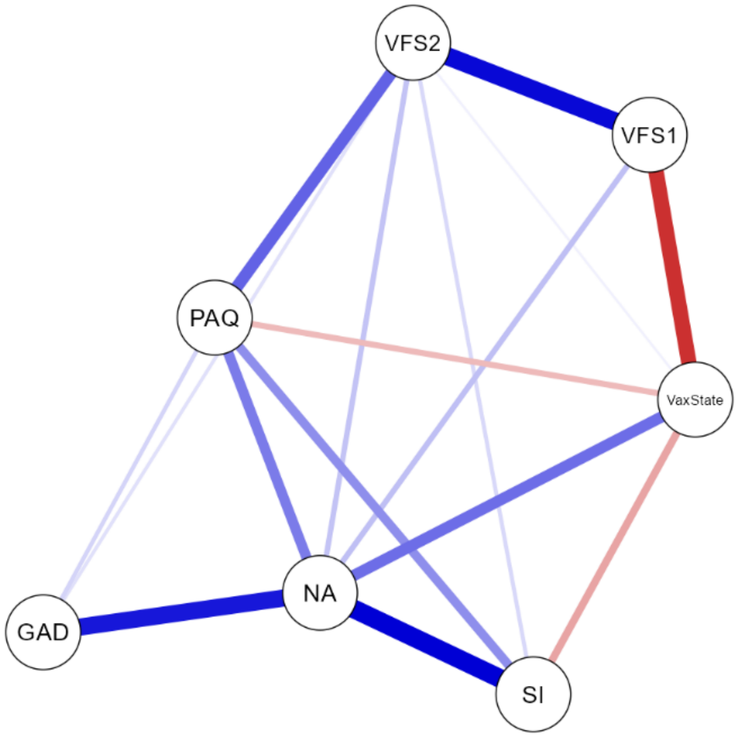

2.Female

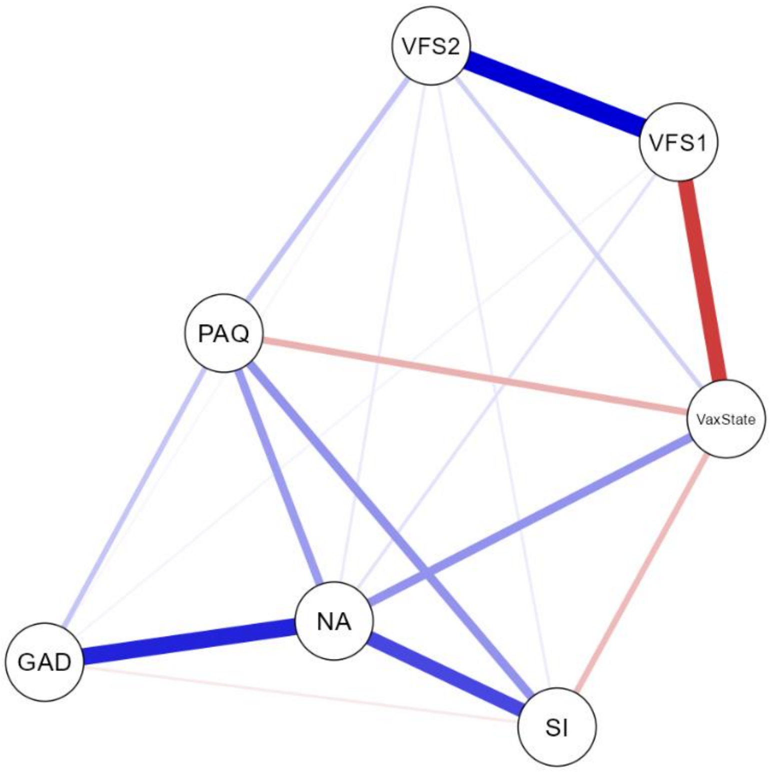

Centrality Plot for male (1)/female (2)

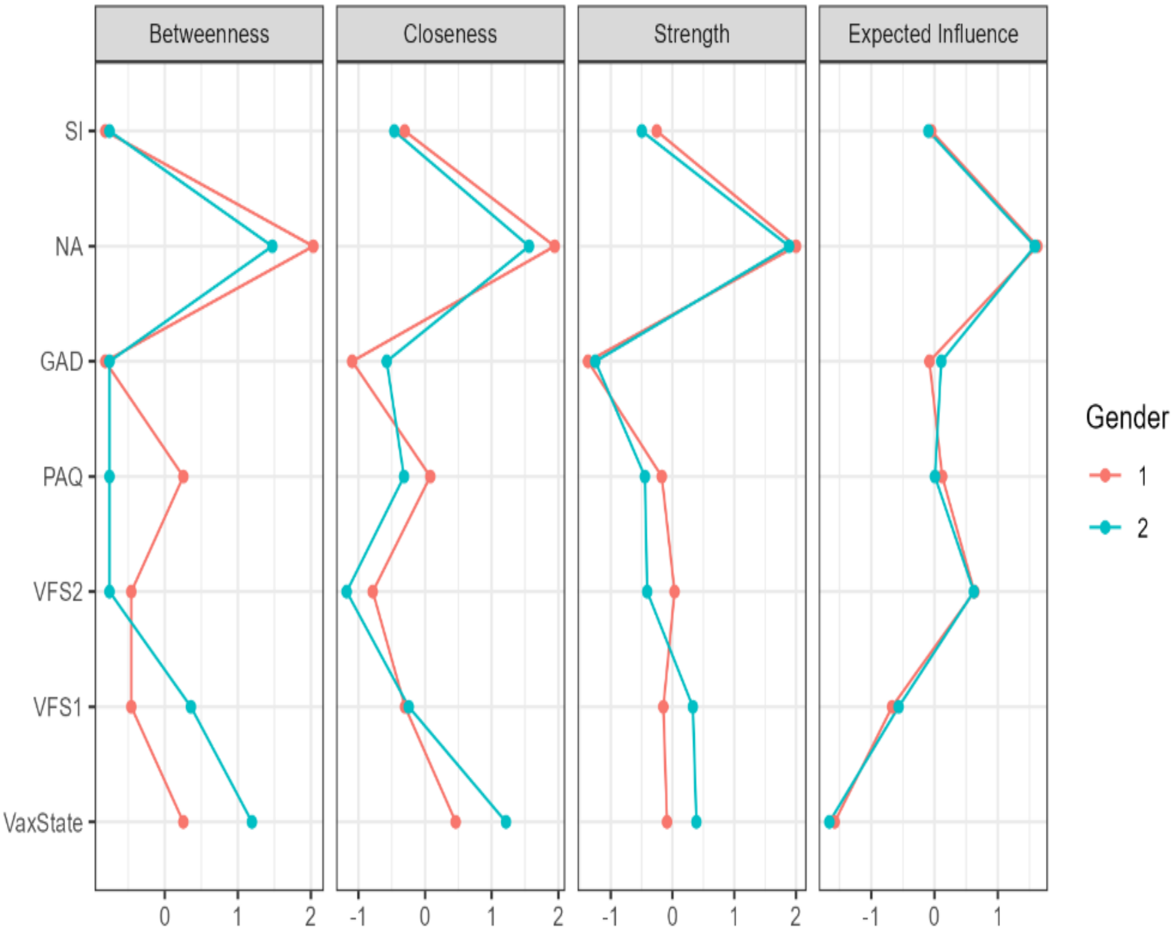

Bootstrap summary of Network

Type	Number of bootstraps
Nonparametric	1000

Edge Stability for male sample

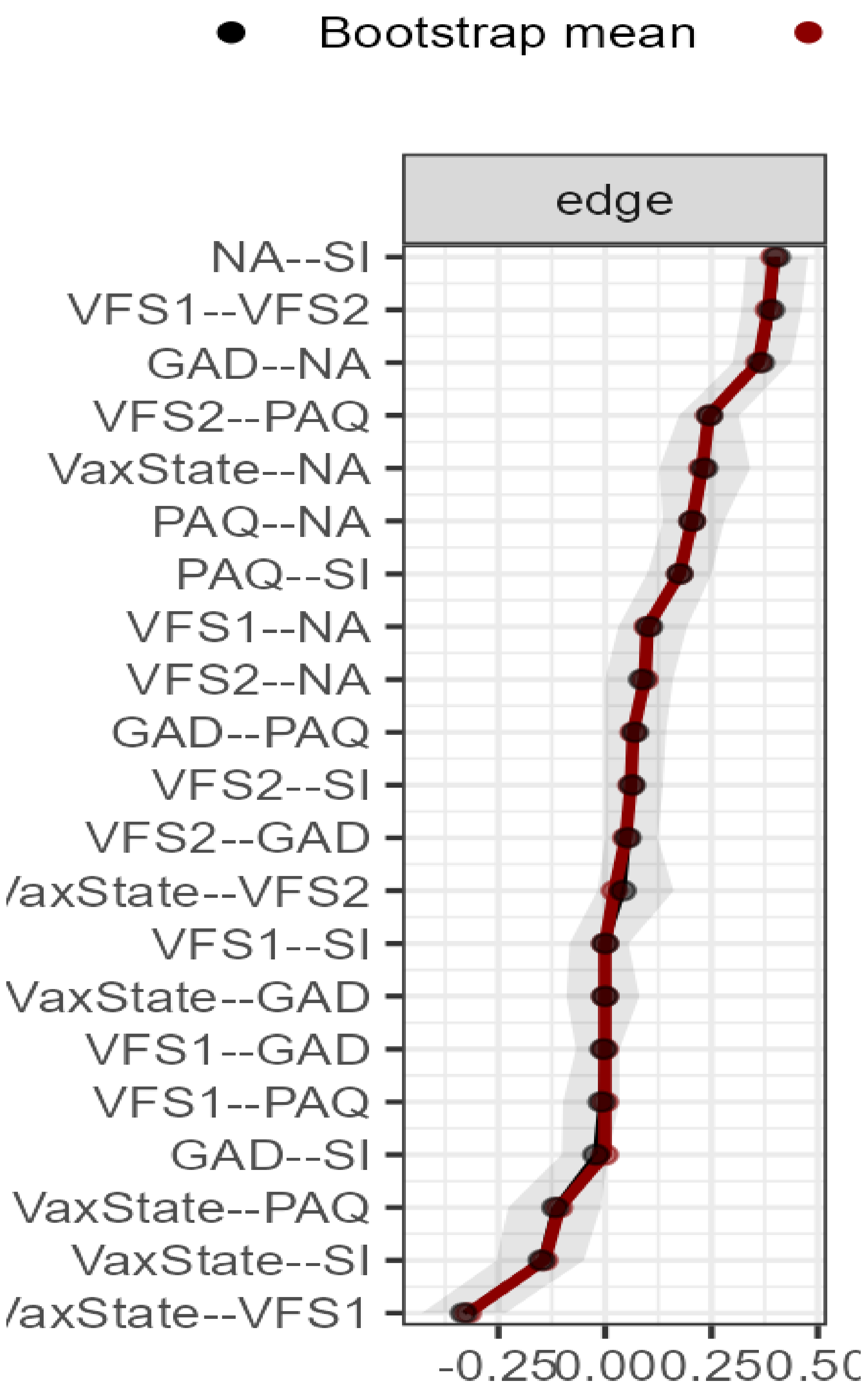

Edge Stability for female sample

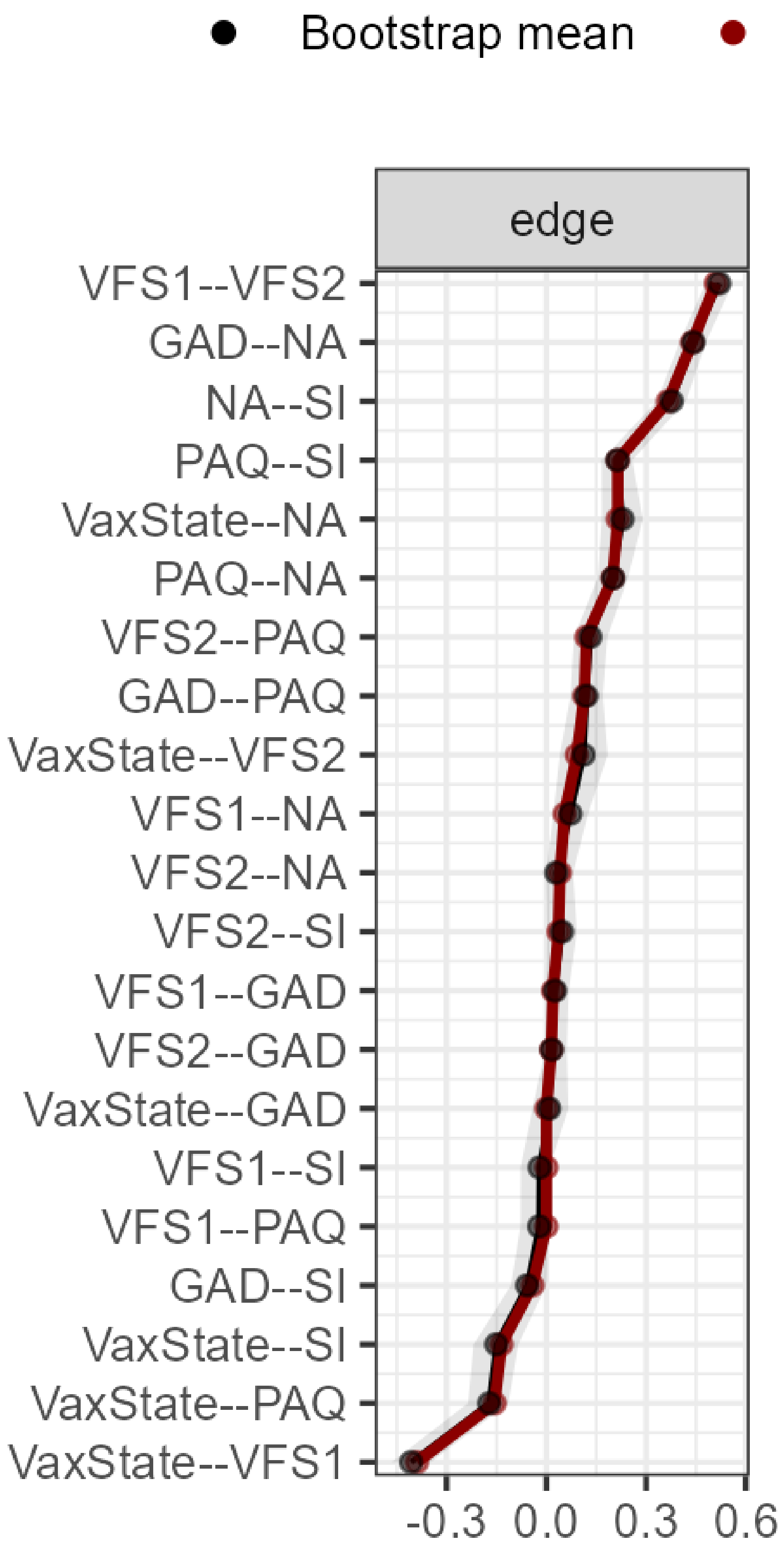

Supplementary material

Article: Links Between Vaccination Fear, Anxiety, Alexithymia and Type D Personality Related Vaccination Decision: A Network Analysis in a Multicultural Sample

Centrality Stability for male sample

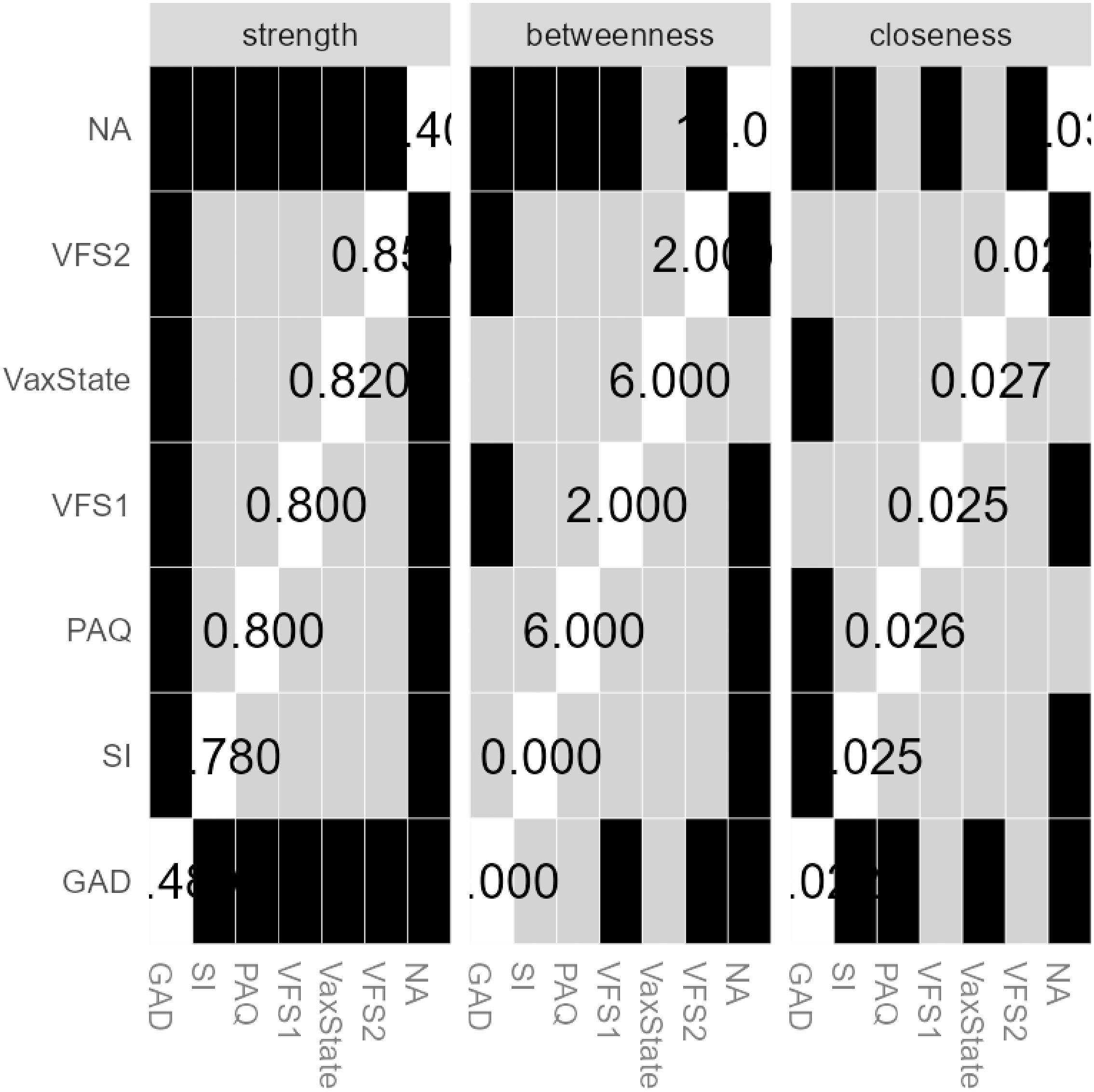

Supplementary material

Article: Links Between Vaccination Fear, Anxiety, Alexithymia and Type D Personality Related Vaccination Decision: A Network Analysis in a Multicultural Sample

Centrality Stability for female sample

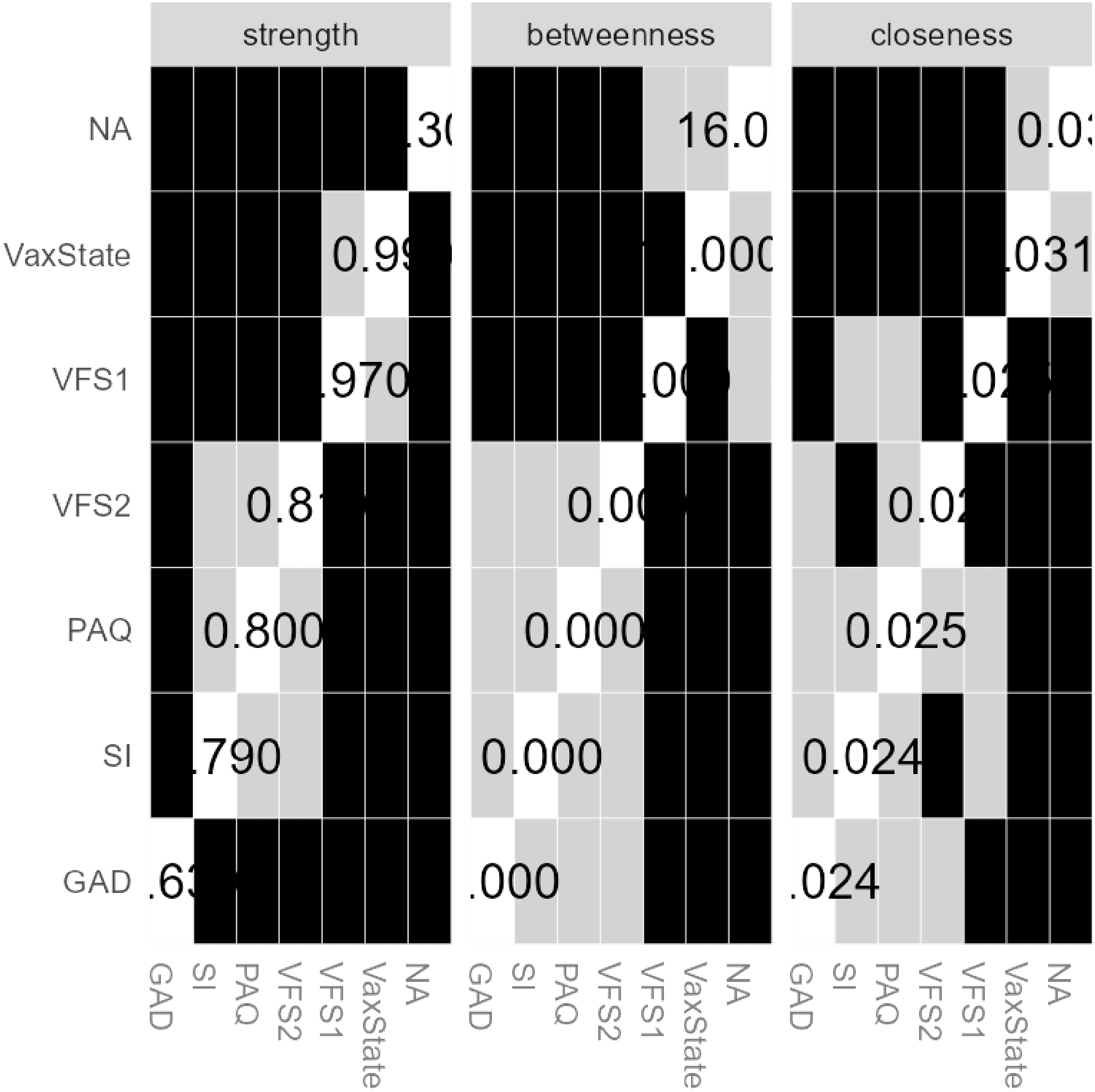

2.3. Network Analysis for the different countries under análisis

Summary of Network

Network	Number of nodes	Number of non-zero edges	Sparsity
1.Spain	7	16 / 21	0.238
2.Italy	7	16 / 21	0.238
3.Lebanon	7	16 / 21	0.238
4.Nigeria	7	12 / 21	0.429
5.Turkey	7	13 / 21	. 0.381
6.Ukraine	7	11 / 21	0.476

Centrality Plots

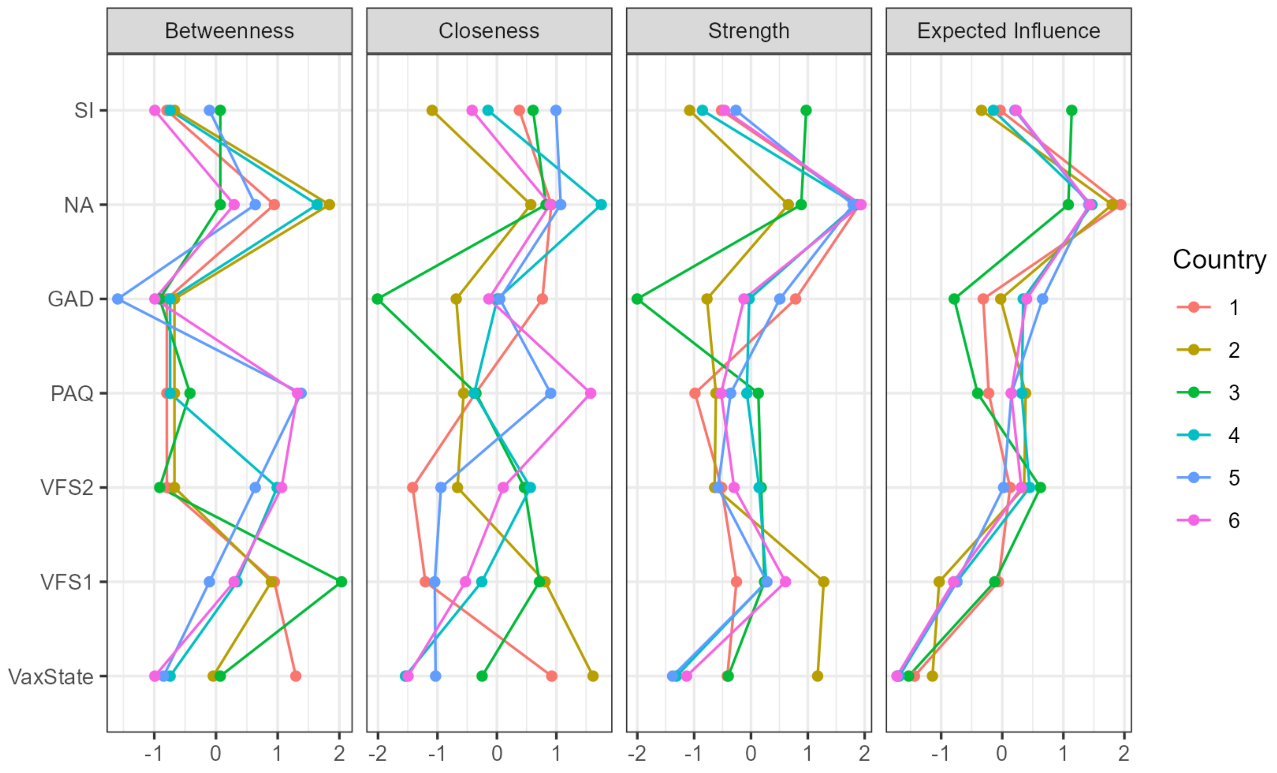

1. Spain

Centrality measures per variable

	Network			
Variable	Betweenness	Closeness	Strength	Expected influence
VaxState	1.294	0.922	-0.417	-1.437
VFS1	0.946	-1.204	-0.255	-0.064
VFS2	-0.796	-1.417	-0.517	0.128
GAD	-0.796	0.765	0.786	-0.312
PAQ	-0.796	-0.359	-0.985	-0.222
NA	0.946	0.915	1.907	1.941
SI	-0.796	0.378	-0.519	-0.034

Weights matrix

	Network						
Variable	VaxState	VFS1	VFS2	GAD	PAQ	NA	SI
VaxState	0.000						
VFS1	-0.067	0.000					
VFS2	0.035	0.573	0.000				
GAD	-0.215	0.051	0.000	0.000			
PAQ	0.000	0.030	0.000	0.112	0.000		
NA	0.193	0.000	0.026	0.581	0.213	0.000	
SI	0.167	0.000	0.018	-0.028	0.177	0.262	0.000

Supplementary material

Article: Links Between Vaccination Fear, Anxiety, Alexithymia and Type D Personality Related Vaccination Decision: A Network Analysis in a Multicultural Sample

Network plot

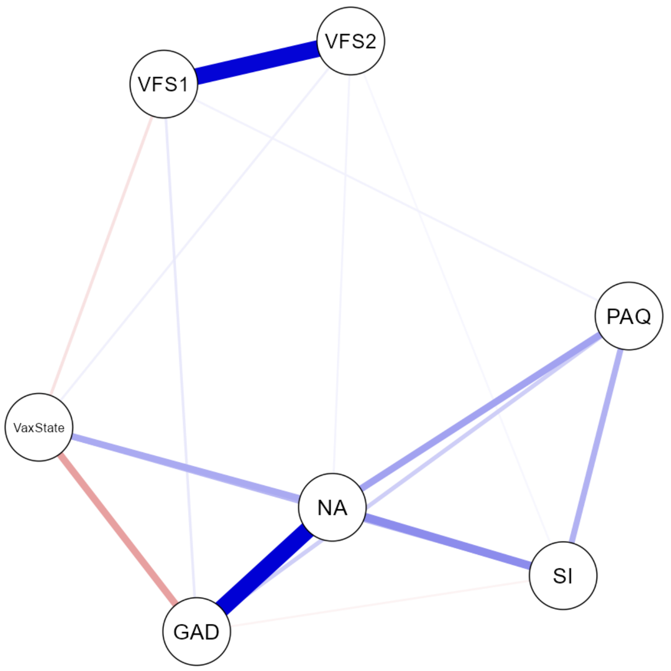

Centrality Plot

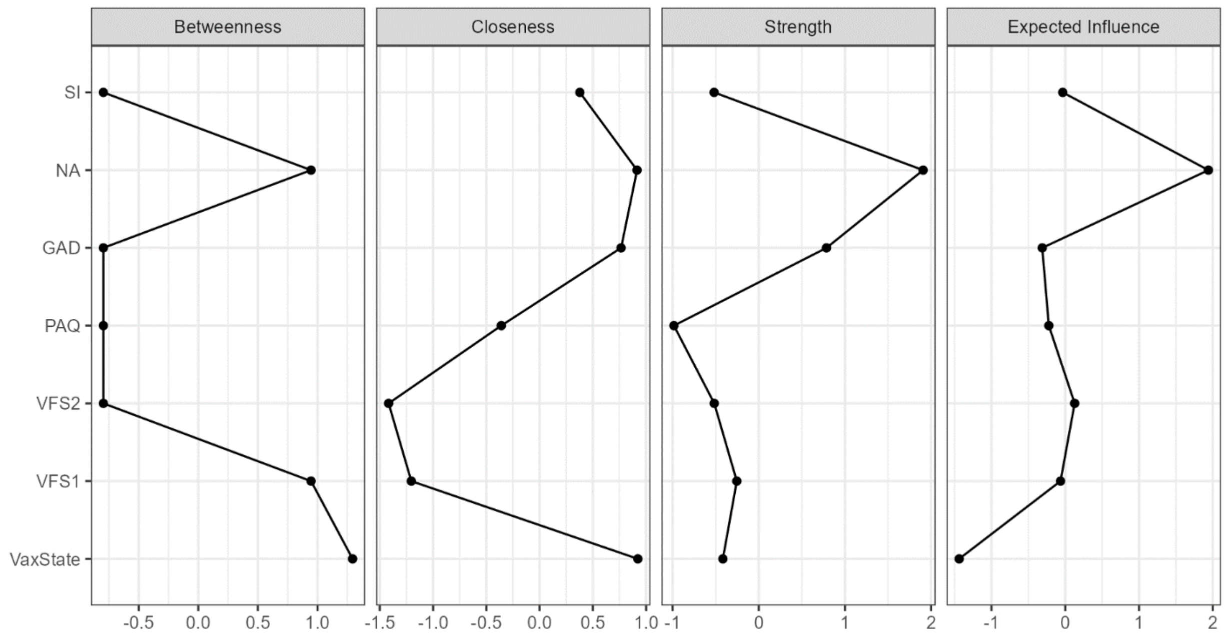

Bootstrap summary of Network

Type	Number of bootstraps
Nonparametric	1000

Edge Stability

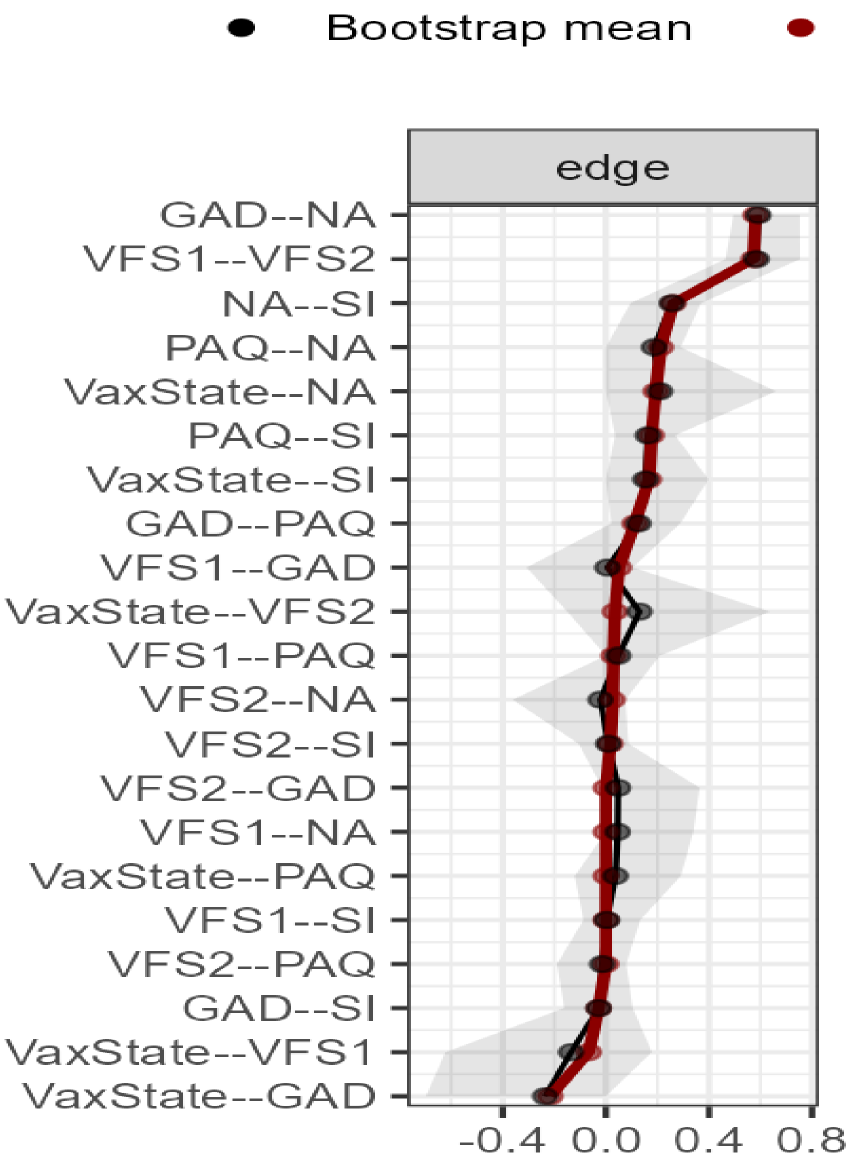

Supplementary material

Article: Links Between Vaccination Fear, Anxiety, Alexithymia and Type D Personality Related Vaccination Decision: A Network Analysis in a Multicultural Sample

Centrality Stability

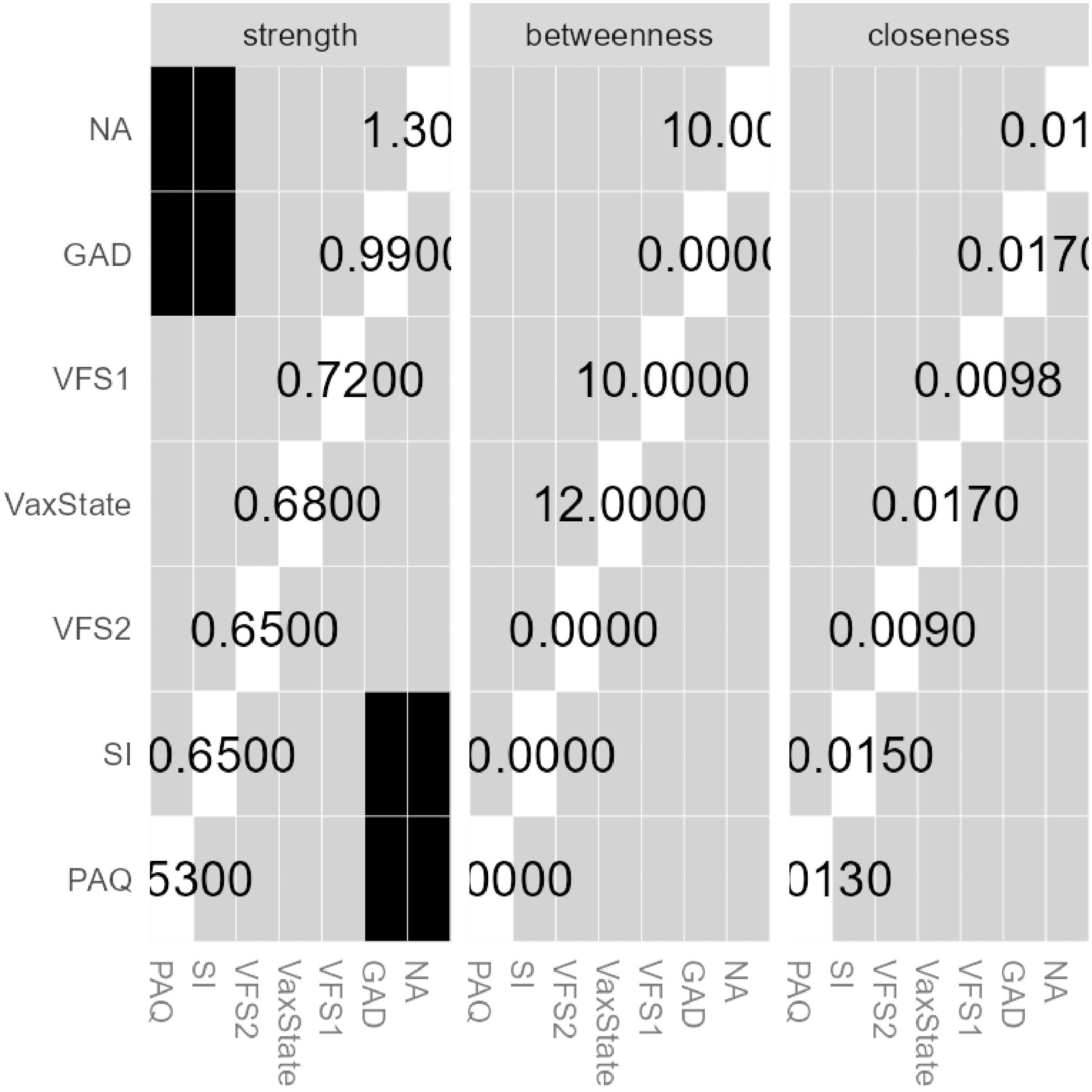

2. Italy

Centrality measures per variable

	Network			
Variable	Betweenness	Closeness	Strength	Expected influence
VaxState	-0.045	1.617	1.173	-1.143
VFS1	0.897	0.810	1.280	-1.032
VFS2	-0.673	-0.663	-0.640	0.358
GAD	-0.673	-0.684	-0.774	-0.025
PAQ	-0.673	-0.560	-0.617	0.382
NA	1.839	0.570	0.657	1.799
SI	-0.673	-1.089	-1.079	-0.339

Weights matrix

	Network						
Variable	VaxState	VFS1	VFS2	GAD	PAQ	NA	SI
VaxState	0.000						
VFS1	-0.731	0.000					
VFS2	0.245	0.539	0.000				
GAD	0.032	0.000	0.000	0.000			
PAQ	0.285	0.208	-0.022	0.034	0.000		
NA	0.197	0.160	0.000	0.607	0.115	0.000	
SI	0.101	0.000	0.000	-0.074	0.151	0.289	0.000

Supplementary material

Article: Links Between Vaccination Fear, Anxiety, Alexithymia and Type D Personality Related Vaccination Decision: A Network Analysis in a Multicultural Sample

Network plot

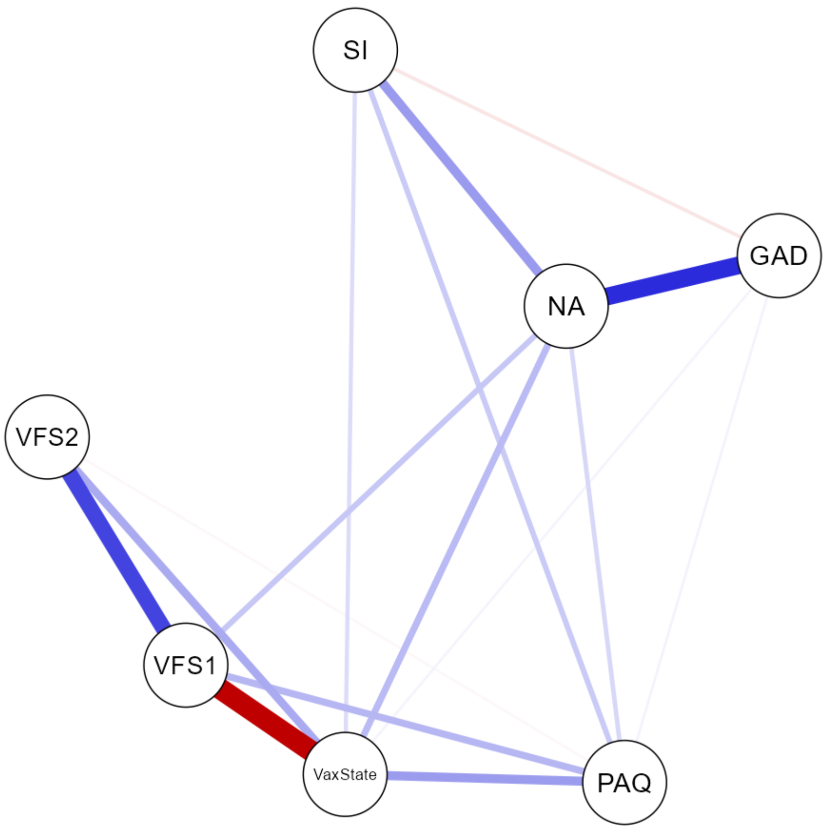

Centrality Plot

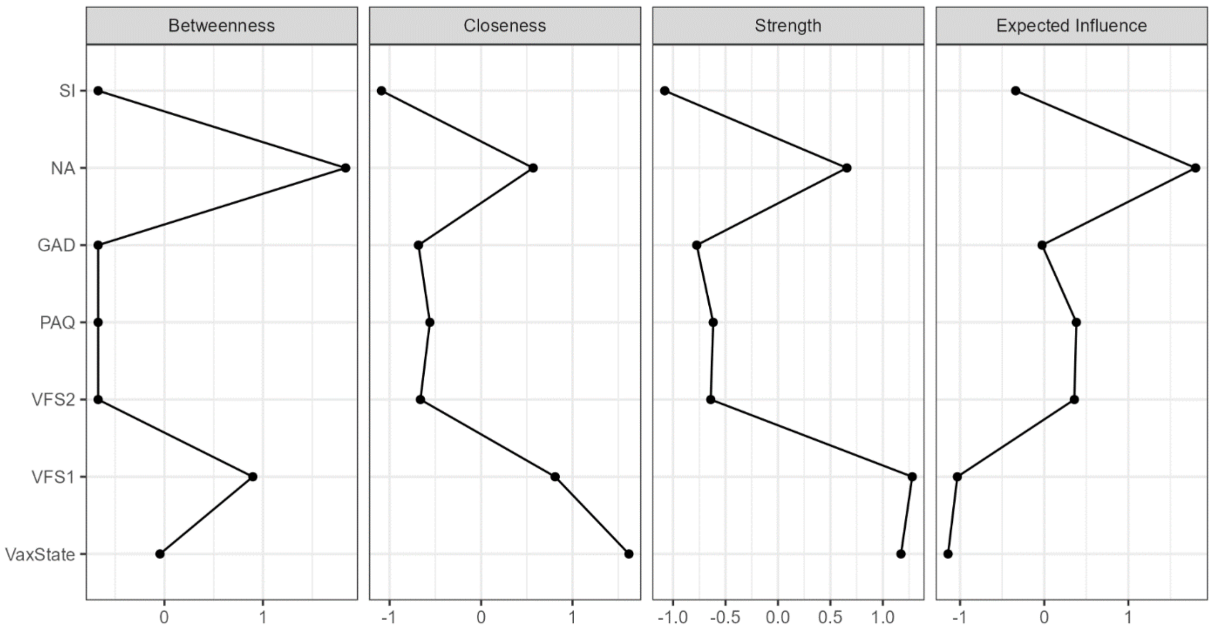

Bootstrap summary of Network

Type	Number of bootstraps
Nonparametric	1000

Edge Stability

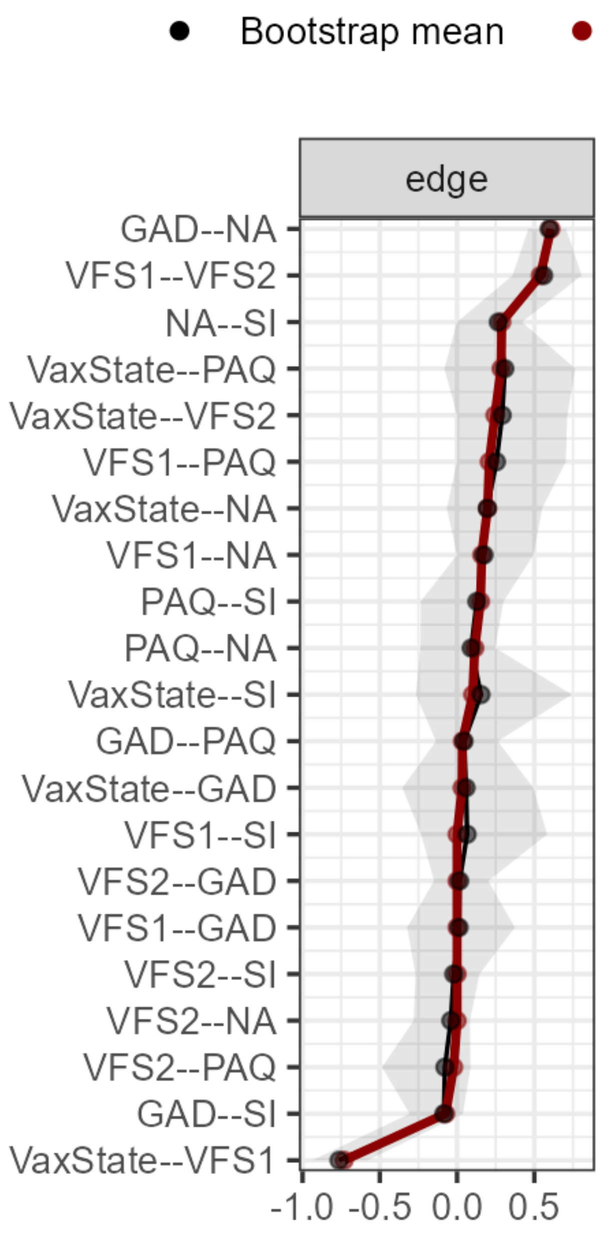

Supplementary material

Article: Links Between Vaccination Fear, Anxiety, Alexithymia and Type D Personality Related Vaccination Decision: A Network Analysis in a Multicultural Sample

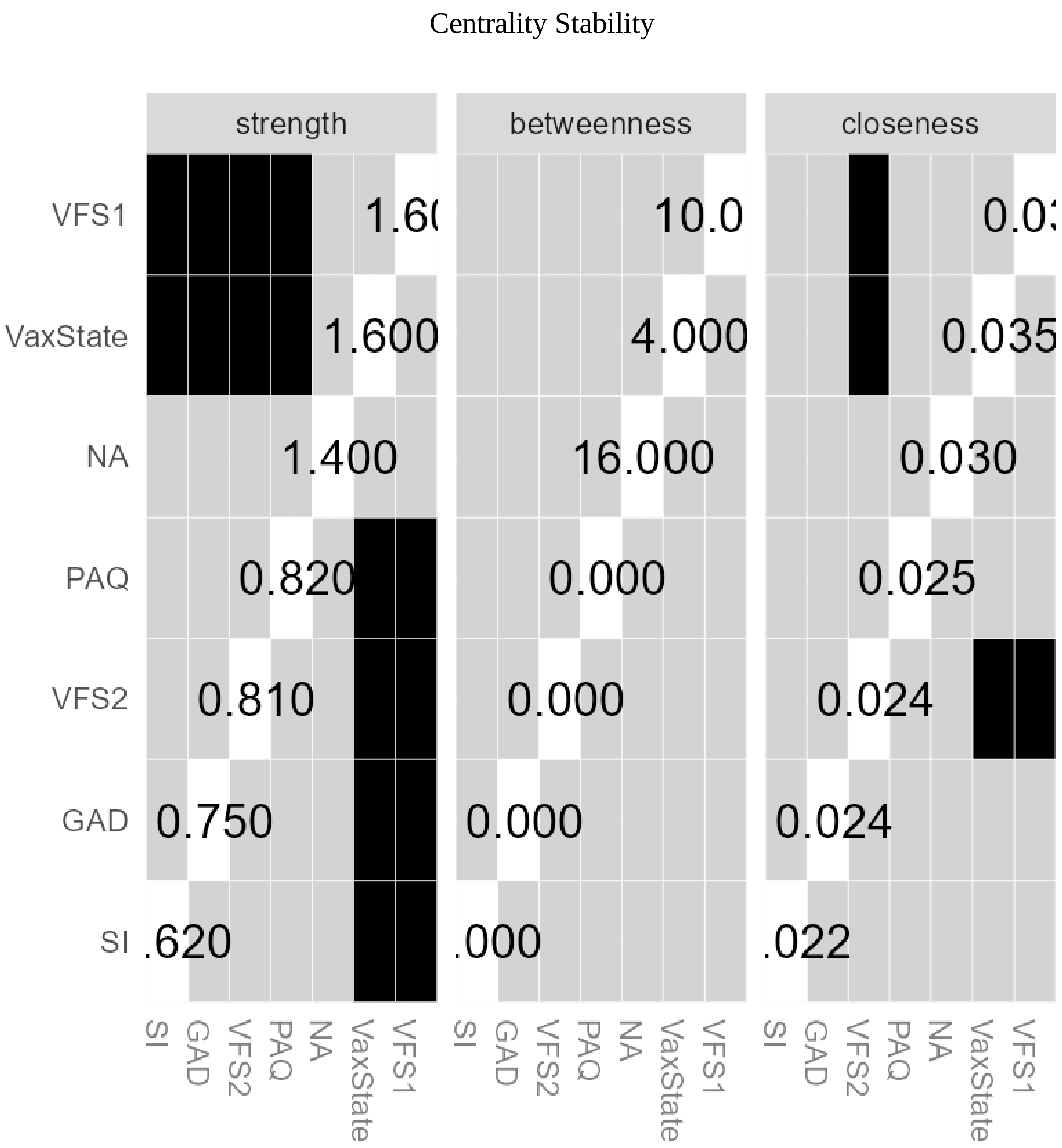

3. Lebanon

Centrality measures per variable

	Network			
Variable	Betweenness	Closeness	Strength	Expected influence
VaxState	-0.886	-1.006	-0.875	-1.379
VFS1	1.771	0.747	0.101	-0.060
GAD	-0.886	-1.467	-1.450	-0.733
PAQ	-0.221	0.158	0.186	-0.084
NA	0.443	0.887	1.088	1.180
SI	-0.221	0.681	0.950	1.076

Weights matrix

	Network						
Variable	VaxState	VFS1	VFS2	GAD	PAQ	NA	SI
VaxState	0.000						
VFS1	-0.201	0.000					
VFS2	0.077	0.315	0.000				
GAD	0.000	0.113	0.072	0.000			
PAQ	-0.255	0.052	0.108	0.000	0.000		
NA	0.160	0.245	0.004	0.000	0.196	0.000	
SI	0.023	0.000	0.331	0.000	0.280	0.536	0.000

Supplementary material

Article: Links Between Vaccination Fear, Anxiety, Alexithymia and Type D Personality Related Vaccination Decision: A Network Analysis in a Multicultural Sample

Network plot

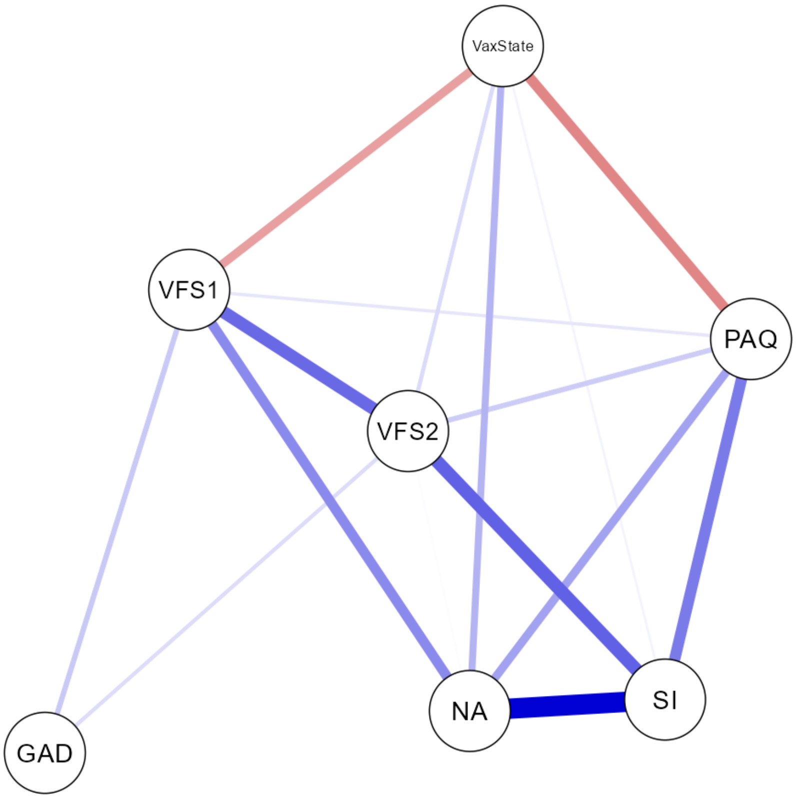

Centrality Plot

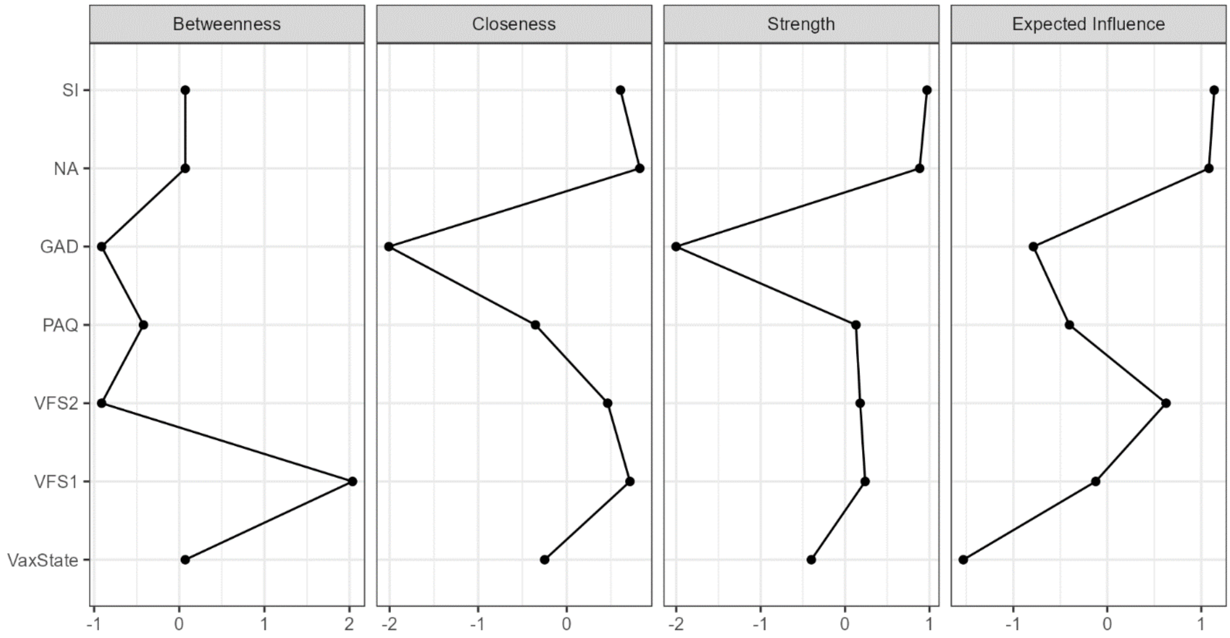

Supplementary material

Article: Links Between Vaccination Fear, Anxiety, Alexithymia and Type D Personality Related Vaccination Decision: A Network Analysis in a Multicultural Sample

Bootstrap summary of Network

Type	Number of bootstraps
Nonparametric	1000

Edge Stability

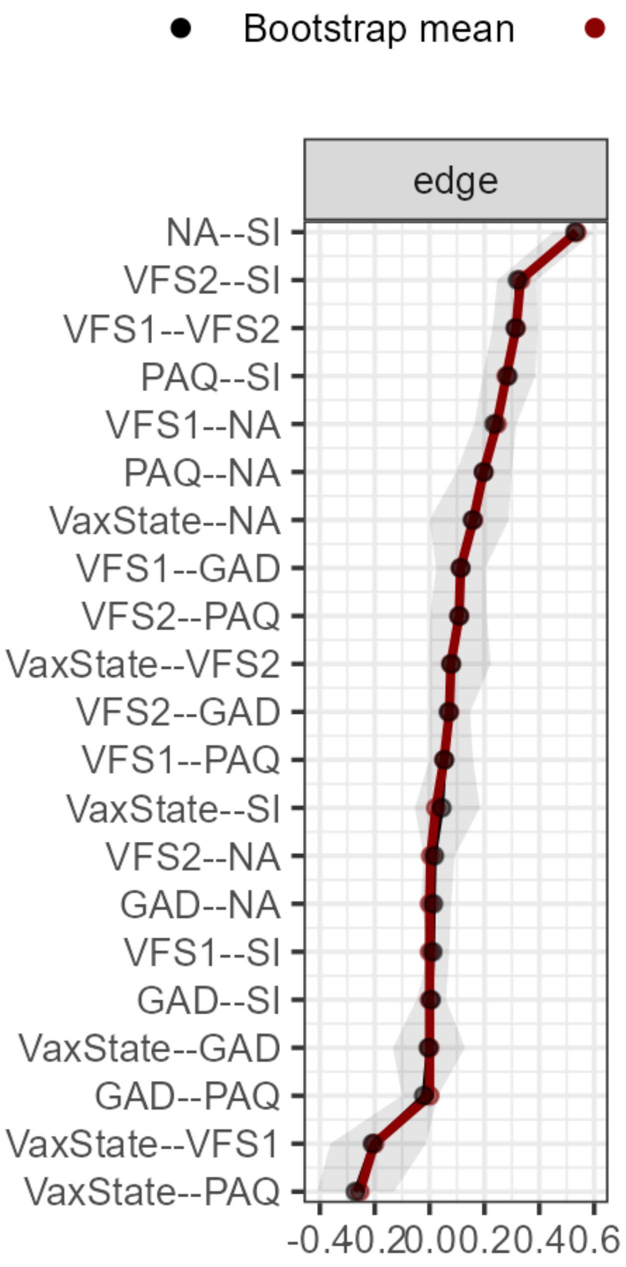

Centrality Stability

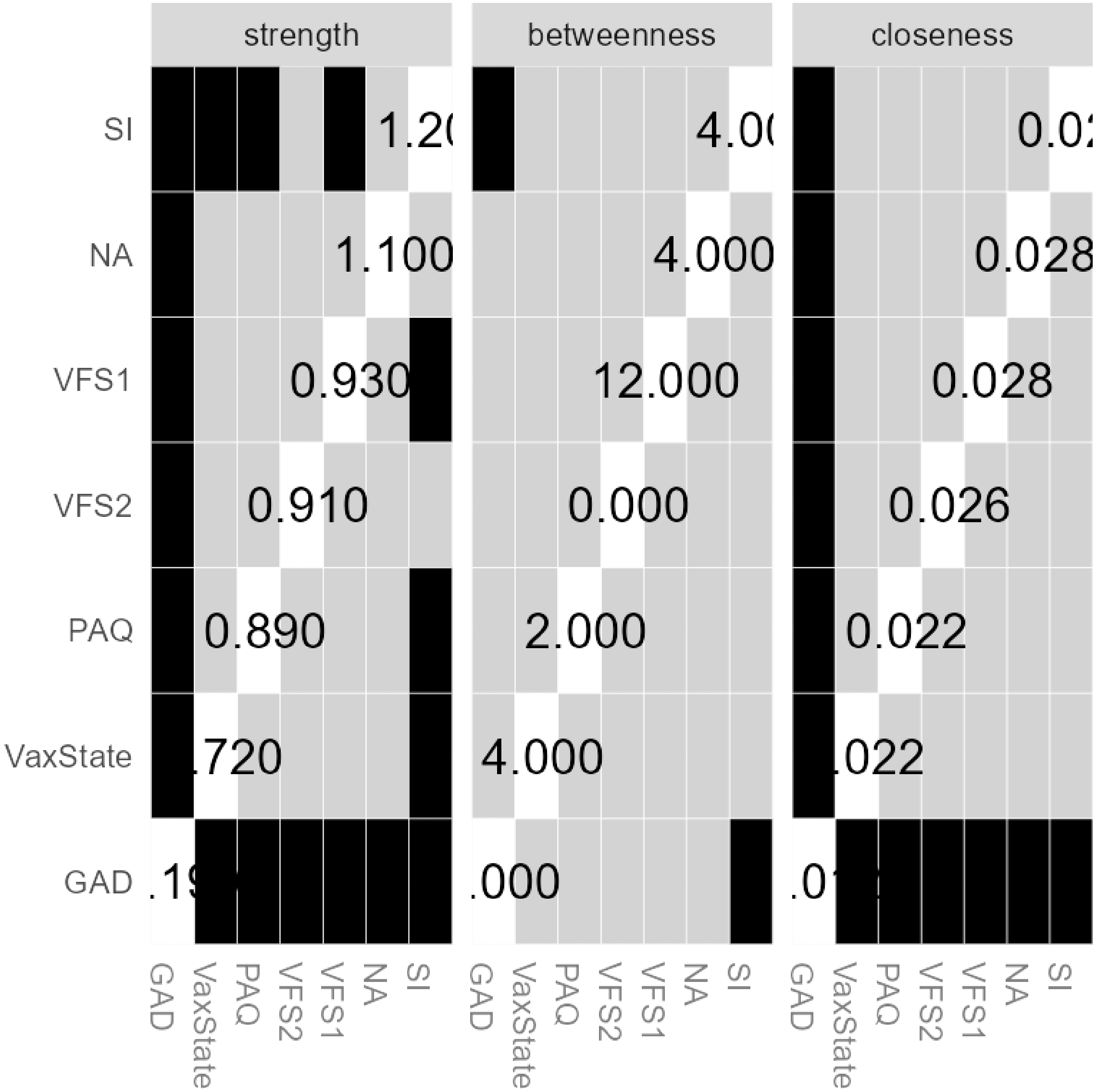

3. Nigeria

Centrality measures per variable

Variable	Network			
	Betweenness	Closeness	Strength	Expected influence
VaxState	-0.848	-1.434	-1.074	-1.307
VFS1	1.187	-0.886	-0.530	-0.992
GAD	-0.848	0.248	0.190	0.453
PAQ	0.170	1.062	0.123	0.414
NA	1.187	0.984	1.803	1.389
SI	-0.848	0.026	-0.513	0.044

Weights matrix

Variable	Network						
	VaxState	VFS1	VFS2	GAD	PAQ	NA	SI
VaxState	0.000						
VFS1	-0.271	0.000					
VFS2	0.000	0.386	0.000				
GAD	0.000	0.000	0.051	0.000			
PAQ	0.000	0.016	0.083	0.179	0.000		
NA	0.000	0.000	0.121	0.350	0.271	0.000	
SI	0.000	0.000	0.000	0.016	0.036	0.335	0.000

Supplementary material

Article: Links Between Vaccination Fear, Anxiety, Alexithymia and Type D Personality Related Vaccination Decision: A Network Analysis in a Multicultural Sample

Network plot

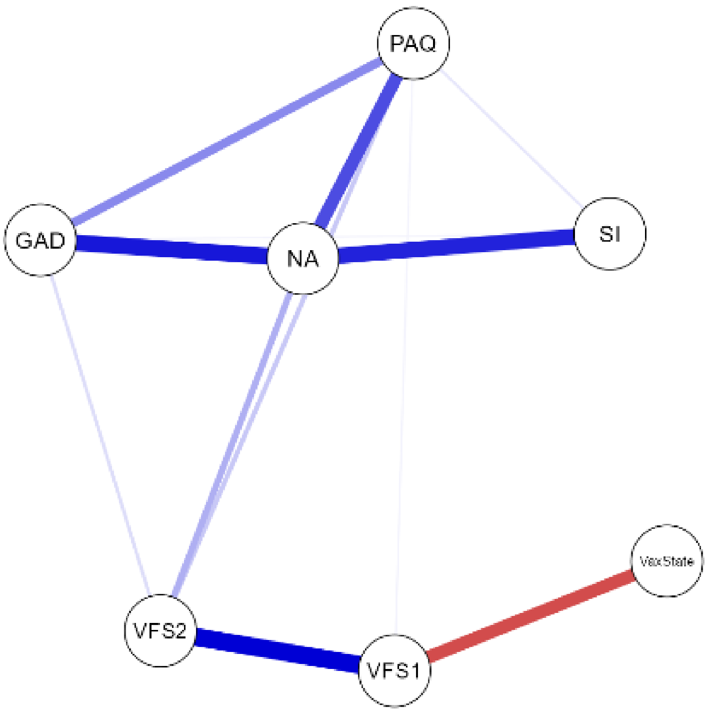

Centrality Plot

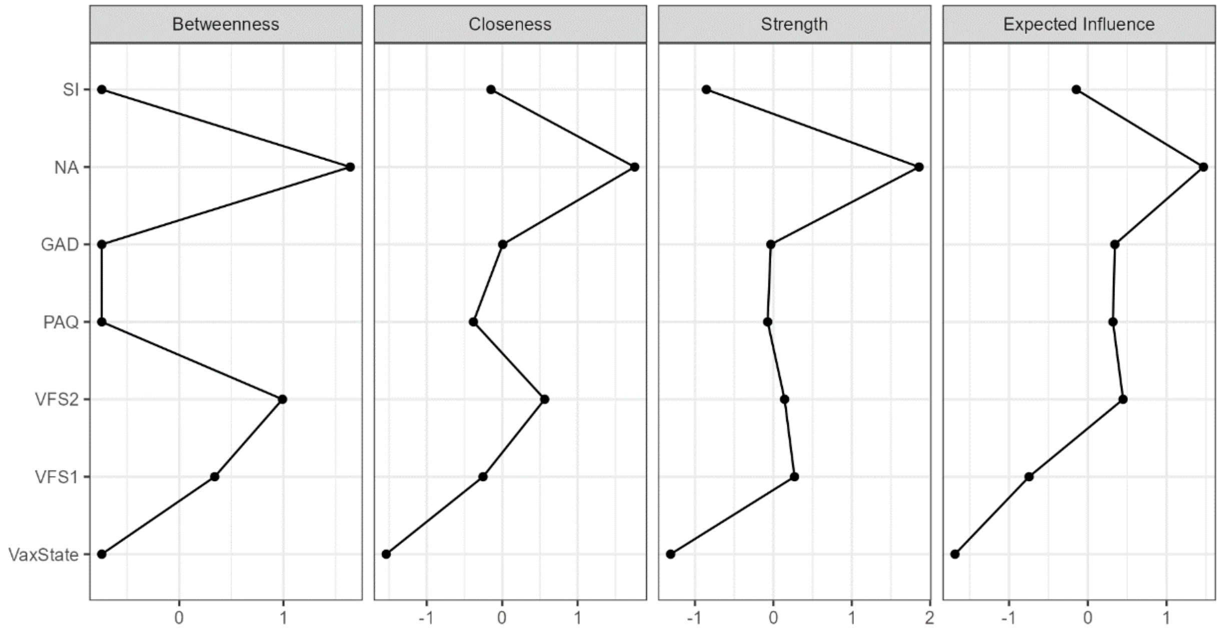

Bootstrap summary of Network

Type	Number of bootstraps
Nonparametric	1000

Edge Stability

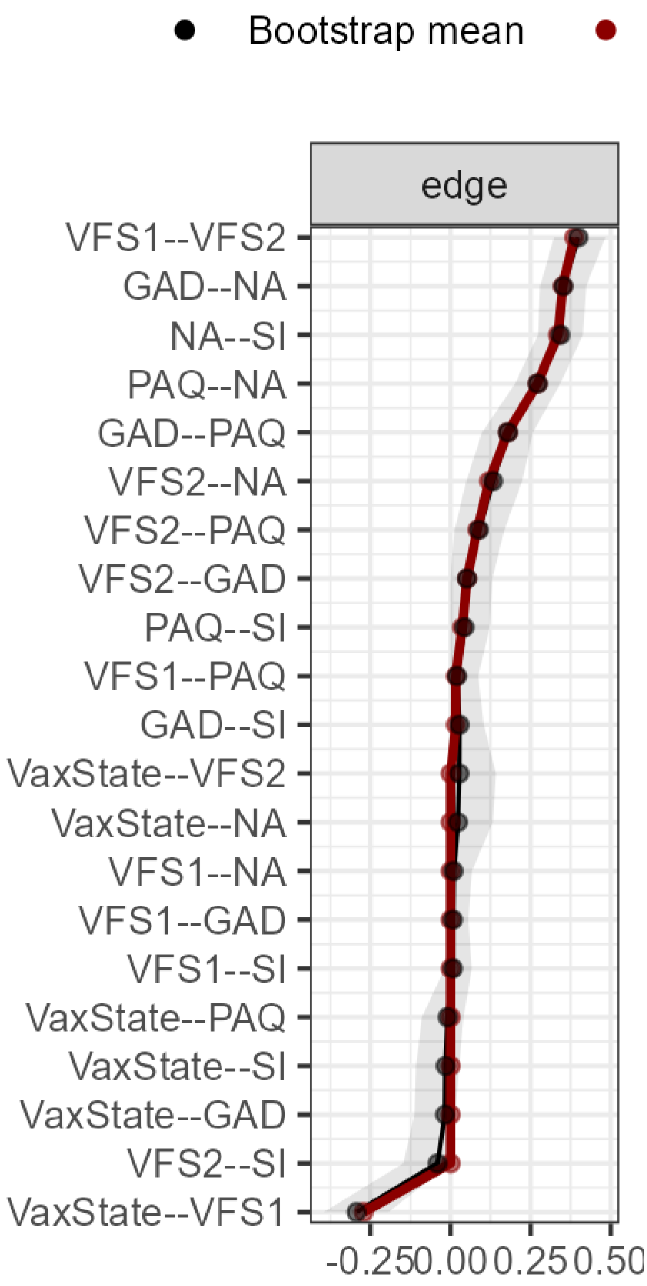

Supplementary material

Article: Links Between Vaccination Fear, Anxiety, Alexithymia and Type D Personality Related Vaccination Decision: A Network Analysis in a Multicultural Sample

Centrality Stability

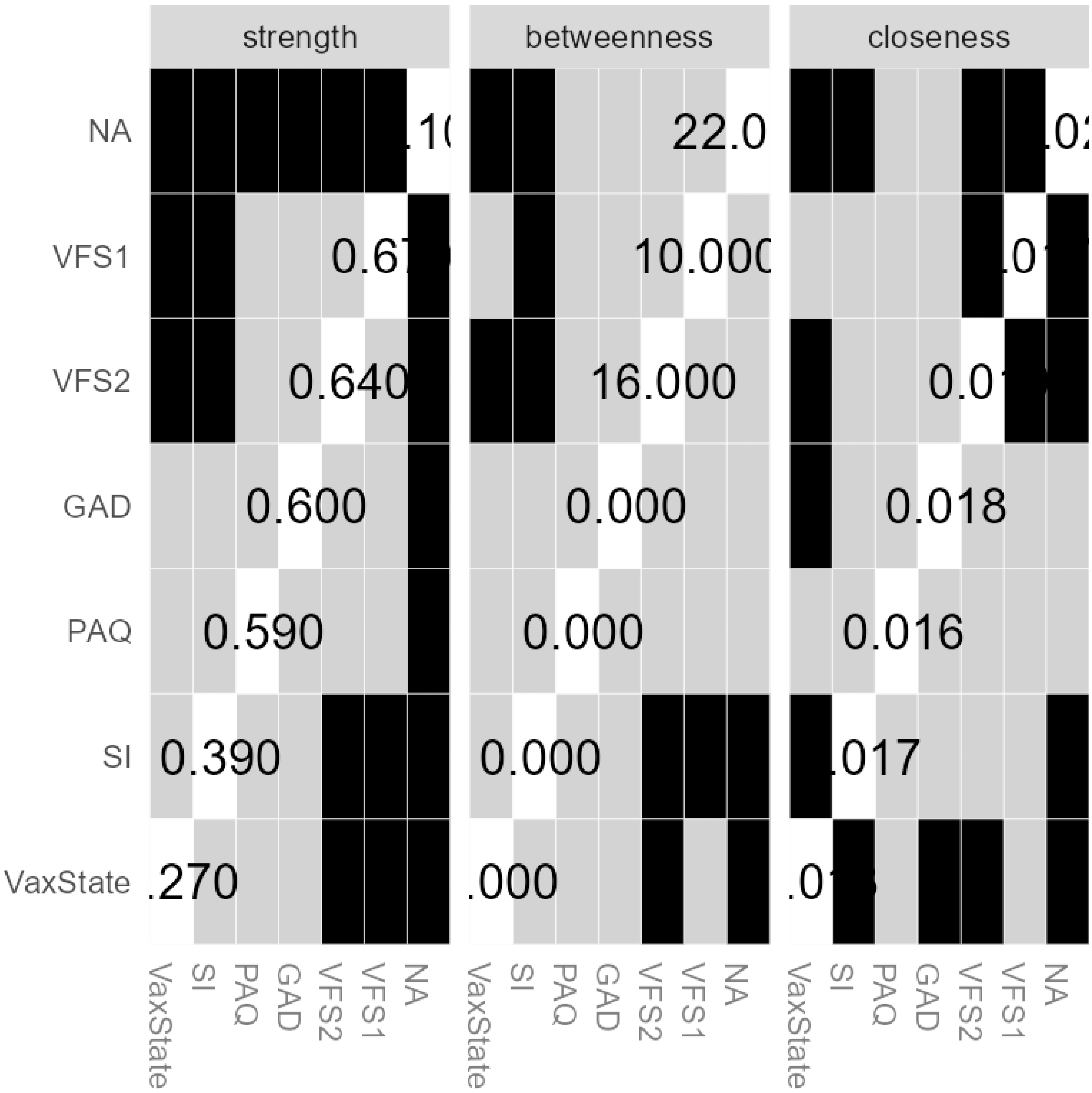

5. Turkey

Centrality measures per variable

Variable	Network			
	Betweenness	Closeness	Strength	Expected influence
VaxState	0.253	-0.774	-0.889	-1.204
VFS1	-0.760	-1.264	-0.850	-1.184
GAD	-0.760	0.825	0.618	0.691
PAQ	-0.760	-0.530	-0.403	0.166
NA	1.772	1.275	1.706	1.251
SI	0.253	0.467	-0.181	0.280

Weights matrix

Variable	Network						
	VaxState	VFS1	VFS2	GAD	PAQ	NA	SI
VaxState	0.000						
VFS1	-0.225	0.000					
VFS2	0.000	0.398	0.000				
GAD	0.037	0.061	0.000	0.000			
PAQ	0.000	0.000	0.103	0.133	0.000		
NA	0.087	0.010	0.000	0.510	0.081	0.000	
SI	0.000	0.000	0.014	0.000	0.244	0.322	0.000

Supplementary material

Article: Links Between Vaccination Fear, Anxiety, Alexithymia and Type D Personality Related Vaccination Decision: A Network Analysis in a Multicultural Sample

Network Plot

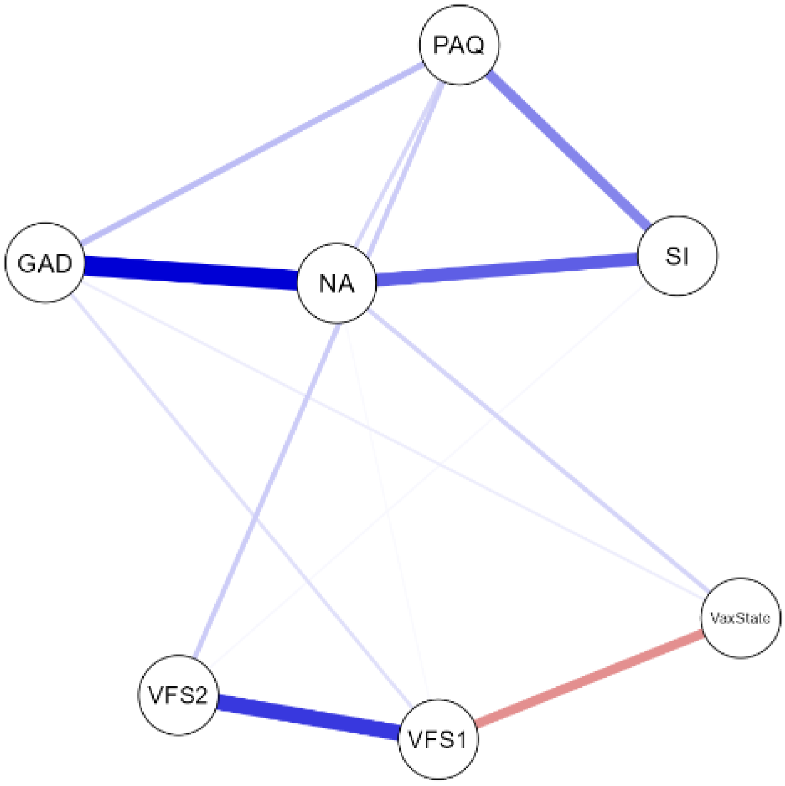

Centrality Plot

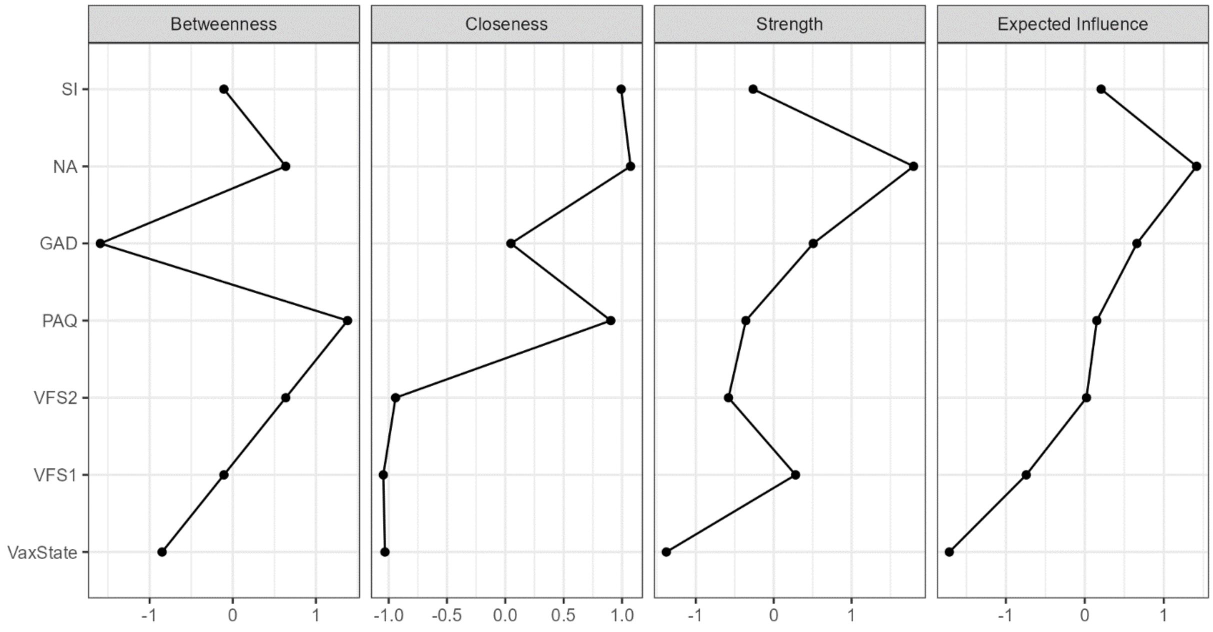

Supplementary material

Article: Links Between Vaccination Fear, Anxiety, Alexithymia and Type D Personality Related Vaccination Decision: A Network Analysis in a Multicultural Sample

Bootstrap summary of Network

Type	Number of bootstraps
Nonparametric	1000

Edge Stability

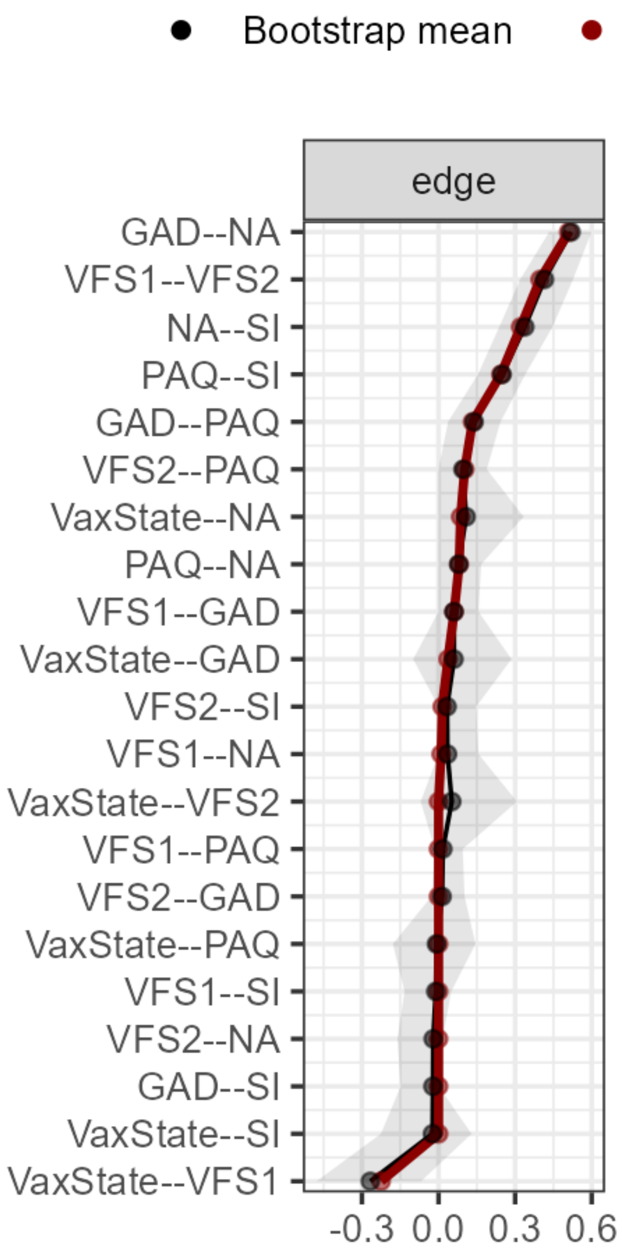

Supplementary material

Article: Links Between Vaccination Fear, Anxiety, Alexithymia and Type D Personality Related Vaccination Decision: A Network Analysis in a Multicultural Sample

Centrality Stability

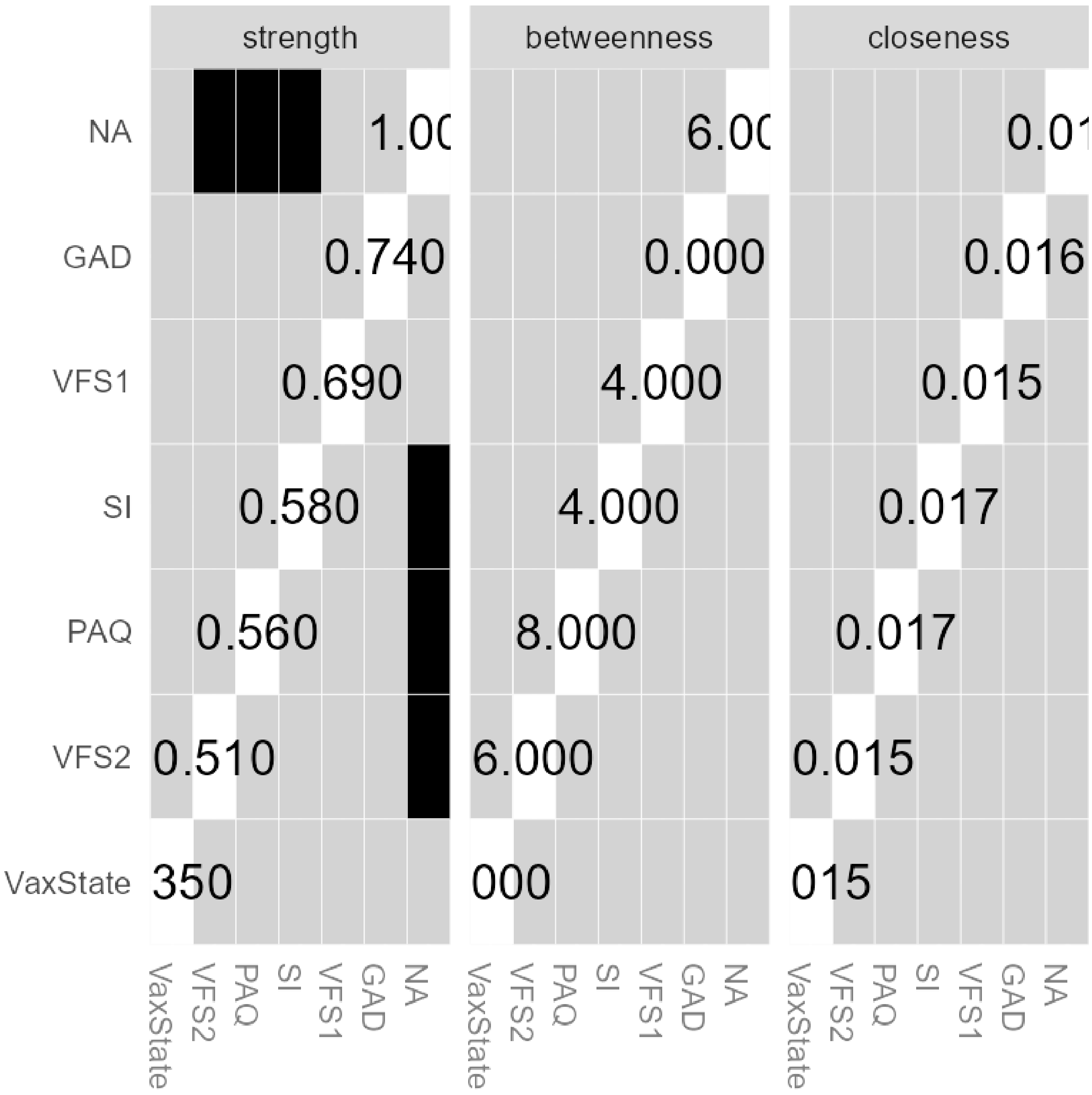

6. Ukraine

Centrality measures per variable

Variable	Network			
	Betweenness	Closeness	Strength	Expected influence
VaxState	0.627	-1.048	-0.471	-1.238
VFS1	-0.878	-1.374	-0.729	-1.143
GAD	-0.878	0.290	0.279	0.636
PAQ	1.379	0.996	-0.507	0.133
NA	0.627	0.951	1.917	1.275
SI	-0.878	0.184	-0.489	0.336

Weights matrix

Variable	Network						
	VaxState	VFS1	VFS2	GAD	PAQ	NA	SI
VaxState	0.000						
VFS1	-0.358	0.000					
VFS2	0.000	0.413	0.000				
GAD	0.000	0.000	0.000	0.000			
PAQ	-0.012	0.000	0.077	0.108	0.000		
NA	0.000	0.000	0.034	0.495	0.188	0.000	
SI	0.000	0.000	0.038	0.000	0.125	0.361	0.000

Supplementary material

Article: Links Between Vaccination Fear, Anxiety, Alexithymia and Type D Personality Related Vaccination Decision: A Network Analysis in a Multicultural Sample

Network plot

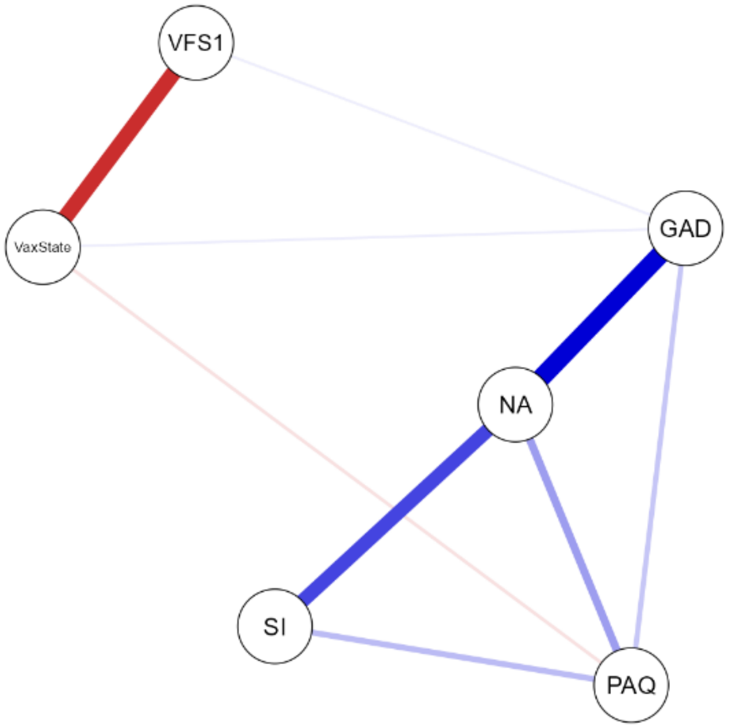

Centrality Plot

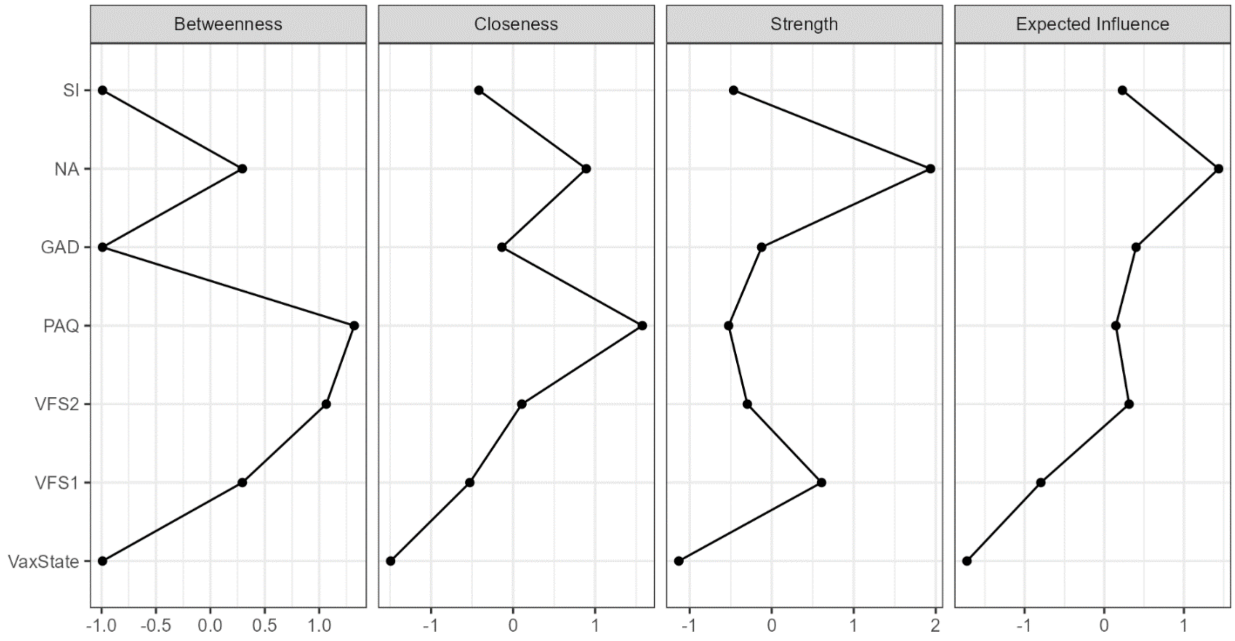

Supplementary material

Article: Links Between Vaccination Fear, Anxiety, Alexithymia and Type D Personality Related Vaccination Decision: A Network Analysis in a Multicultural Sample

Bootstrap summary of Network

Type	Number of bootstraps
Nonparametric	1000

Edge Stability

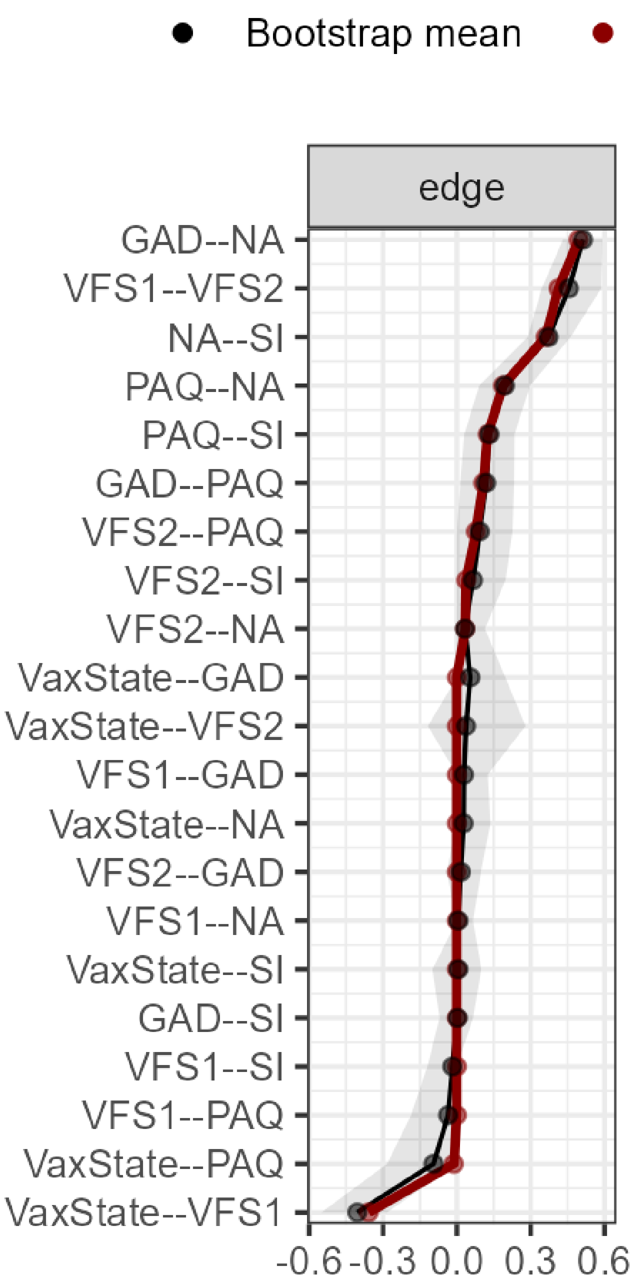

Supplementary material

Article: Links Between Vaccination Fear, Anxiety, Alexithymia and Type D Personality Related Vaccination Decision: A Network Analysis in a Multicultural Sample

Centrality Stability

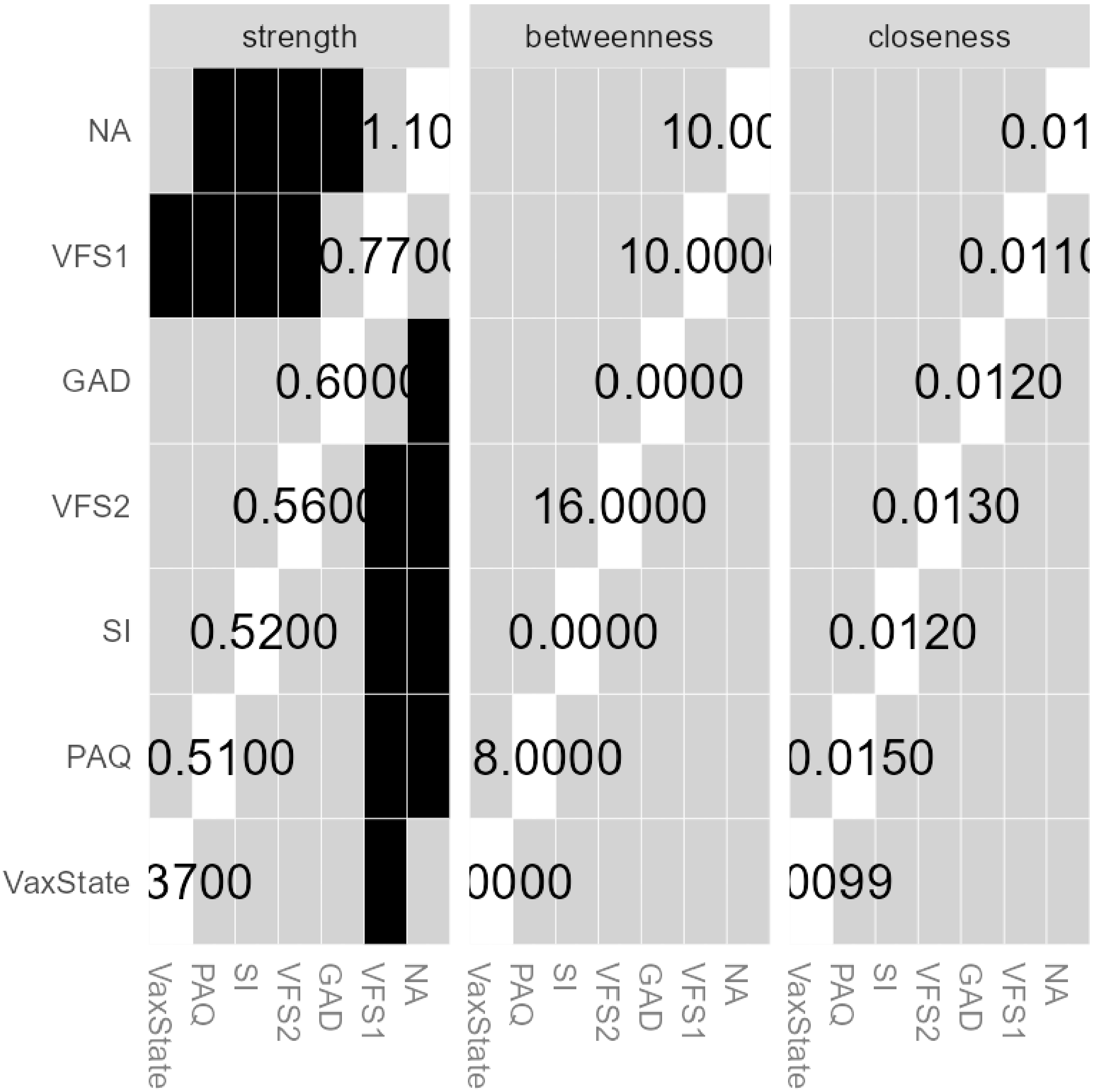

Supplement: Supplementary file 1 [file behavsci-14-00761-s001.zip › behavsci-3081481-supplementary.pdf]
